# Opposing Association of Lung Neutrophils and PD‐L1^+^ Monocytes in Age‐Related Severity of SARS‐CoV‐2 Infection

**DOI:** 10.1111/acel.70588

**Published:** 2026-06-12

**Authors:** Serban Morosan, Clémence Granier, Noëlline Guillou, Selma Bennacer, Eléonore Weber‐Delacroix, Julianne Peronnet, François Lanthiez, Sandrine Barthelemy, Christelle Enond, Jean‐Luc Diehl, Elena Paillaud, Carine El‐Sissy, Anne‐Geneviève Marcelin, Vincent Calvez, Stéphane Marot, Amélie Guihot, Christophe Parizot, Karim Dorgham, Guy Gorochov, Christophe Combadière, Alexandre Boissonnas

**Affiliations:** ^1^ Department of Exact Sciences “Ion Ionescu de la Brad” Iasi University of Life Sciences Iași Romania; ^2^ UMS28, Phenotypage du Petit Animal Sorbonne Université/INSERM Paris France; ^3^ Hémato‐Immunology Unit, CHU d'Orléans, UMR 1364 INTHERNA, LI2RSO Université d'Orléans Orléans France; ^4^ Inserm U1135, CNRS ERL 8255, Centre d'Immunologie et Des Maladies Infectieuses (CIMI‐Paris) Sorbonne Université Paris France; ^5^ Inserm U955, Institut Mondor de Recherche biomédicale (IMRB) Université Paris‐Est Créteil Créteil France; ^6^ Medical Intensive Care Department Assistance Publique Hôpitaux de Paris (AP‐HP) Georges Pompidou European Hospital Paris France; ^7^ Inserm, The Paris Cardiovascular Research Center UMR U970, Team: Endotheliopathy and Hemostasis Disorders, Paris Cité University Paris France; ^8^ Geriatric Department Assistance Publique Hôpitaux de Paris (AP‐HP), Georges Pompidou European Hospital Paris France; ^9^ Department of Immunology Assistance Publique, Hôpitaux de Paris (AP‐HP), Georges Pompidou European Hospital Paris France; ^10^ University of Paris Cité Paris France; ^11^ Inflammation, Complement and Cancer Team, Cordeliers Research Center Institut National de la Santé et de la Recherche Médicale (INSERM) Unité Mixte de Recherche UMRS1138 Paris France; ^12^ COMET “Complement Expertise and Therapeutics” Fédération Hospitalo‐Universitaire Paris France; ^13^ Inserm, Institut Pierre Louis d'Epidémiologie et Santé Publique (IPLESP), Assistance Publique‐Hôpitaux de Paris (AP‐HP), Hôpital Pitié‐Salpêtrière, Service de Virologie Sorbonne Université Paris France; ^14^ Département d'Immunologie Assistance Publique Hôpitaux de Paris (AP‐HP) Hôpital Pitié Salpêtrière Paris France

**Keywords:** aging, COVID‐19, infection severity, monocytes, neutrophils

## Abstract

Advanced age is a major determinant of adverse outcomes during acute infections, yet the immunological mechanisms by which aging alters immune regulation and shapes disease trajectories remain poorly understood. Using SARS‐CoV‐2 infection as a model of acute viral challenge, we investigated how aging alters myeloid responses in the lungs. Across infected mouse models and human cohorts, disease severity was associated with a pronounced shift in myeloid balance, characterized by an increased neutrophil‐to‐monocyte ratio. Neutrophils exhibited prolonged retention within the pulmonary microvasculature and formed large co‐aggregates with monocytes. In parallel, severe disease was associated with a defect in classical monocyte activation in the lungs, notably through reduced PD‐L1 upregulation. This defect was not observed in the circulation, indicating tissue‐dependent dysregulation of inflammatory and regulatory markers. Aging further accentuated this imbalance. Both aged mice and elderly patients displayed a reduced proportion of PD‐L1‐expressing lung monocytes, despite enhanced pulmonary neutrophil recruitment. This age‐associated alteration distinguished severe from non‐severe disease and characterized a maladaptive myeloid response to acute viral challenge. Together, these findings identify impaired, compartment‐specific myeloid immune regulation as a central feature of age‐related vulnerability to severe infection. They highlight that tissue‐dependent regulatory failure compounds inflammatory excess as a key determinant of disease outcome and suggest that restoring myeloid regulatory function may offer therapeutic benefit in older individuals during acute respiratory infections.

## Graphical abstract

Aging skews pulmonary myeloid responses during acute SARS‐CoV‐2 infection toward severe disease. Disease progression is characterized by excessive lung neutrophil accumulation together with impaired activation of classical monocytes, including a defect in PD‐L1 upregulation. This opposing imbalance distinguishes non‐critical from critical disease and underlies age‐related vulnerability to severe respiratory infection.

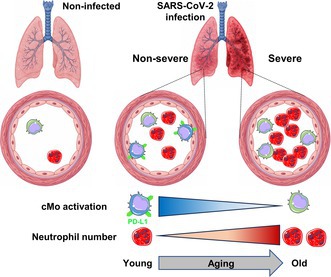

## 1 Introduction

Aging is associated with immunosenescence, characterized by declining adaptive immune function, and inflammaging, a state of chronic low‐grade inflammation driven by lifelong antigenic exposure and cellular stress (Fulop et al. 2017). Aging is also accompanied by a bias in myelopoiesis (Sun et al. 2025) and an increased neutrophil‐to‐lymphocyte ratio (NLR), a robust predictor of disease severity and mortality that reflects neutrophilia and impaired adaptive immune responses (Li et al. 2015; Liu et al. 2020). Severe acute viral infections are characterized by profound immune dysregulation, including lymphopenia, elevated inflammatory markers, and delayed yet excessive activation of the innate immune system (Nguyen et al. 2024). These features mirror age‐associated immune remodeling and compromise effective antiviral immunity.

In the context of COVID‐19, this imbalance favors excessive cytokine production and tissue injury rather than efficient viral clearance, thereby exacerbating disease severity (Qin et al. 2020; Mueller et al. 2020). These mechanisms are particularly pronounced in older individuals and contribute to the exaggerated inflammatory responses observed during severe disease (O'Driscoll et al. 2021; Zinatizadeh et al. 2023).

Consistent with these mechanistic insights, epidemiological analyzes from large cohorts identify advanced age as one of the strongest independent risk factors for hospitalization, progression to acute respiratory distress syndrome, and death following SARS‐CoV‐2 infection (Zhou et al. 2020; Williamson et al. 2020). Even after adjustment for comorbidities, older individuals account for a disproportionate burden of severe disease and mortality, indicating that intrinsic age‐related immune alterations critically shape disease outcomes. Accordingly, COVID‐19 provides a relevant model for studying severe viral infections in elderly individuals.

The myeloid compartment, particularly neutrophils and monocytes, plays a central role in COVID‐19 pathogenesis (Schulte‐Schrepping et al. 2020). In severe COVID‐19, neutrophils exhibiting an immature profile show disproportionate expansion and activation, contributing to endothelial damage, immunothrombosis, and organ dysfunction (Barnes et al. 2020; Combadière et al. 2021). Neutrophil‐extracellular trap (NET) formation has been strongly linked to acute respiratory distress syndrome (ARDS) and microvascular complications in COVID‐19 patients, with elevated neutrophil counts and NET‐associated markers correlating with poor clinical outcomes (Hazeldine and Lord 2021). In elderly individuals, age‐related defects in neutrophil regulation further amplify these pathological responses, promoting sustained inflammation and tissue injury (Middleton et al. 2020).

Beyond neutrophils, profound alterations in the monocyte compartment have been consistently observed in severe COVID‐19 (Knoll et al. 2021; Rong et al. 2025). Single‐cell transcriptomic and flow cytometric analyzes have revealed a dysregulated myeloid landscape characterized by the expansion of inflammatory monocyte subsets, reduced antigen‐presenting capacity, and impaired interferon signaling (Schulte‐Schrepping et al. 2020; Giamarellos‐Bourboulis et al. 2020; Silvin et al. 2020). In particular, classical and intermediate monocytes exhibit heightened inflammatory profiles and are preferentially recruited to inflamed tissues, including the lungs, where they contribute to cytokine production and immunopathology (Zhang, Wang, et al. 2020; Sánchez‐Cerrillo et al. 2020). However, in a mouse model, CCR2‐dependent monocyte recruitment has been shown to exert a protective effect by regulating viral load and cytokine responses (Vanderheiden et al. 2021). Collectively, these findings indicate that severe COVID‐19, particularly in the elderly, is driven by a complex interplay between neutrophil hyperactivation and monocyte dysregulation. Understanding the relative contribution of these subsets to pathogenesis is essential for the development of targeted immunomodulatory strategies aimed at reducing morbidity and mortality, particularly in elderly individuals.

Here, we investigated the dynamics of neutrophil and monocyte subset accumulation in the lungs following SARS‐CoV‐2 infection in a mouse model using in situ live imaging and flow cytometry, and we assessed their relative contribution to age‐related disease severity in both mice and infected patients.

## 2 Material and Methods

### 2.1 Mice

B6.Cg‐Tg(K18‐ACE2)2Prlmn/J mice (ACE2) were purchased from Jackson Laboratory (RRID:IMSR_JAX:034860) and intercrossed with Cx3cr1egfp/+ (Jung et al. 2000), Csf1r‐Gal4VP16/UAS‐ECFP (Ovchinnikov et al. 2008), and Csf1r‐mApple (Hawley et al. 2018) to generate the ACE2‐RGB‐mac mouse (Petit et al. 2024). All mice were bred and aged at the UMS28 animal facility, Pitié‐Salpêtrière. Adult mice were 4 ± 1 months old; old mice were 24 ± 2 months old at the time of infection. All mice were maintained under specific pathogen–free conditions at 22°C and monitored up to twice daily after SARS‐CoV‐2 inhalation. All experimental protocols were approved by the local ethics committee for animal research and validated by the French Ministry under the number APAFIS#28776‐2020121811451196. Sample sizes were chosen to ensure reproducibility of the experiments and according to the 3Rs (replacement, reduction, and refinement) of animal ethics regulations.

### 2.2 SARS‐CoV‐2 Virus Preparation

The SARS‐CoV‐2 strain used (B1 lineage, Genbank: MW322968) was isolated from a COVID‐19 patient hospitalized at the Pitié‐Salpêtrière University Hospital in March 2020. Viral stocks were amplified through one or two passages in Vero cells (ATCC CCL‐81). Supernatants were harvested on day 4, filtered, ultracentrifuged, and resuspended in HNE buffer. The virus titer was determined in Vero E6 cells (ATCC CRL‐1586) by a limiting dilution assay allowing calculation of a tissue culture infective dose around 50%. Viral stock was then titrated in vivo (see Section 2.3) to reach an intermediate phenotype with a lethal dose close to 50% (LD50). In vivo titration was performed at least three times during the whole study and established at 0.31 plaque forming units (PFU) according to our stock solution.

### 2.3 Infection With SARS‐CoV‐2 Virus

Mice were infected with SARS‐CoV‐2 and maintained in an animal biosafety level 3 (ABSL3) containment laboratory and studied at indicated days post‐infection (dpi). Mice were kept five per cage, thereby allowing potential cross‐infection. Infection rate was tested in three independent experiments by RT‐PCR on lung tissues 7 days post‐infection. Viral copies were detected in 87.5% of tested mice with high variability in their number. In one case out of 32, viral copies were undetected despite the presence of severe clinical symptoms.

For infection, mice were anesthetized with a mixture of xylazine/ketamine i.p. (5 mg/kg and 80 mg/kg respectively) and infected intranasally with the corresponding PFU of SARS‐CoV‐2 diluted in sterile PBS (8.5 μL of solution per nostril). After infection, mice's health was monitored over time and scored on appearance and behavior according to the following severity scoring: For appearance: normal scored 0, lack of grooming scored 1, dull coat scored 2, and piloerection scored 3. For behavior: tremors scored 0, reduced mobility with stable temperature and breathing scored 1, low mobility with abdominal breathing and stable temperature scored 2, and hypothermia or prostration scored 3. A 20% weight loss was directly scored 5 (Terminal end‐point). Reaching a severity score above 2 was considered clinically severe, while keeping a clinical status of a maximum of 2 was considered clinically not severe.

### 2.4 RT‐PCR for SARS‐CoV‐2 Detection

Pulmonary tissues (2–3 mm^3^ pieces) were homogenized with 600 μL of NUCLISENS lysis buffer (Biomerieux) in a TissueLyser LT instrument (Qiagen) for 10 min at 30 Hz and centrifuged at 1500 rpm for 5 min. RNA was extracted from 300 μL of the supernatant with a Nuclisens EasyMAG instrument (Biomerieux) according to the manufacturer's instructions. SARS‐CoV‐2 RNA was quantified using the RealStar SARS‐CoV‐2 RT‐PCR kit 1.0 (Altona Diagnostics) with an in‐house standard curve on a LightCycler 480 Instrument (Roche). RNA concentrations of the RNA extracts were determined using a QubitTM RNA high sensitivity assay kit (Thermo Fisher Scientific).

### 2.5 Human Blood Samples

#### 2.5.1 Old‐Patient (> 70 Years) Cohort From Poisson et al

Blood counts analysis of absolute neutrophil and monocyte counts from patients already described in (Poisson et al. 2024) was reused for NMR calculations.

Briefly, hospitalized patients were prospectively enrolled between April 24, 2020, and March 19, 2021, at the Georges Pompidou European Hospital (HEGP), Paris, France, during the first and second waves of the COVID‐19 pandemic. The study was approved by the local Ethics Committee (CERAPHP, *Centre Comité d'éthique de la recherche AP‐HP Centre*; IRB registration #00011928).

A total of 104 patients aged over 70 were included: 81 COVID‐19–positive patients (43.2% were males and 56.8% females, with a mean age of 84.7 ± 6.6 years (median = 85.7 years)) and 23 COVID‐19–negative controls (34.8% were males and 65.2% females, with a mean age of 85.6 ± 6.5 years (median = 87.3 years)). Inclusion criteria for the COVID‐19–positive group were age > 70 years, confirmed SARS‐CoV‐2 infection by RT‐PCR on a respiratory sample (nasopharyngeal swab or invasive respiratory sample), and onset of COVID‐19 symptoms within 8 days prior to inclusion. The control group consisted of age‐ and sex‐matched patients hospitalized in the geriatric department for non‐infectious conditions and with a negative SARS‐CoV‐2 RT‐PCR test. Exclusion criteria for controls were any confirmed or active infection. For the entire cohort, patients with hematological malignancies or receiving long‐term immunosuppressive therapy, or patients with missing data were excluded (total 8). Sociodemographic characteristics (age and sex), comorbidities, clinical presentation, and COVID‐19 severity were collected using a secure and standardized electronic case report form (REDCap).

Complete blood counts with absolute neutrophil and monocyte counts were obtained immediately after confirmation of SARS‐CoV‐2 infection and prior to initiation of any COVID‐19–specific treatment, including corticosteroids.

#### 2.5.2 Young‐Patient (< 70 Years) Cohort From Kilercik et al

Blood counts analysis of absolute neutrophil and monocyte counts from an independent younger cohort already described was used for NMR calculations (Kilercik et al. 2021). Thanks to the availability of raw data, people aged < 70 years were selected. This cohort consisted of 70.1% males and 29.9% females, with a mean age of 48.1 ± 12.2 years (median = 48.0 years).

### 2.6 COVID‐19 Severity Classification

Within 48 h after diagnosis, COVID‐19–positive patients were classified according to the World Health Organization (WHO) Clinical Progression Scale adapted for elderly patients and stratified into three severity groups: non‐severe disease (WHO score 2–3, asymptomatic or symptomatic without oxygen requirement), non‐critical disease (WHO score 4–5, oxygen therapy ≥ 3 L/min via nasal cannula or mask), and critical disease (WHO score 6–9, requiring high‐flow oxygen therapy, non‐invasive ventilation, or invasive mechanical ventilation). Patients who met clinical criteria for intensive care unit admission but were not admitted following multidisciplinary decision‐making were classified as having critical COVID‐19.

### 2.7 Human LBA

LBA samples from patients described in Guihot et al. (2022) were reused. Briefly, all patients admitted to the intensive care units (ICUs) of Pitié‐Salpêtrière Hospital between March 2020 and April 2020 (first wave) and between October 2020 and November 2020 (second wave) with laboratory‐confirmed SARS‐CoV‐2 infection were included. SARS‐CoV‐2 infection was documented by real‐time reverse transcription polymerase chain reaction (RT‐PCR) on nasopharyngeal swabs or a lower respiratory tract specimen (tracheal aspirate or bronchoalveolar lavage (BAL)). Only patients requiring invasive mechanical ventilation were included. Fiberoptic bronchoscopy with BAL was performed by the clinician's decision as part of routine care. The procedure consisted of instillation and aspiration of three consecutive syringe volumes (50 mL each) of sterile saline into a distal bronchus during fiberoptic bronchoscopy. The total cell count of BAL was determined using a Malassez cell chamber. BAL was then centrifuged (200 g for 5 min); the cell pellet was diluted in phosphate‐buffered saline. Myeloid flow cytometry phenotyping was performed on residual BAL fluid.

In accordance with the current French legislation, written informed consent was obtained from patients and/or relatives. The study protocol was approved by our institution's ethics committee (Immuno‐ COVID‐REA, CER‐Sorbonne Université, no. CER‐SU‐2020‐31).

### 2.8 In Vivo Mouse Treatments

Anti‐mouse Ly6G (clone 1A8, Bio X Cell, RRID:AB_1107721, catalog #BE0075‐1) or rat IgG2a isotype control, anti‐trinitrophenol (clone 2A3, Bio X Cell, RRID:AB_1107769, catalog #BE0089), and anti‐mouse PD‐L1 (clone 10F.9G2, RRID:AB_10949073, catalog #BE0101) or rat IgG2b isotype control, anti‐keyhole limpet hemocyanin (clone LTF‐2, RRID:AB_1107780, catalog #BE0090) were administered intraperitoneally (300 μg in 100 μL PBS/injection) at 4, 6, and 8 dpi.

Blood/tissue partitioning was performed as previously described (Petit et al. 2024). Mice were injected intravenously with 5 μg anti–CD45 PerCP‐Cy5.5 (clone 30‐F11, BD Biosciences, RRID:AB_394003, catalog #567310) (CD45i.v.). Two minutes after injection, the mice were sacrificed. The lungs were harvested and bathed in a large volume of PBS to remove excess antibodies.

### 2.9 Preparation and Tissue Digestion

Mouse lungs were harvested, cut into small pieces, and enzymatically digested with 0.1 mg/mL DNase I (Roche, catalog #10104159001), 1.5 U/mL Dispase II (Gibco, catalog #17105041), and 200 U/mL Collagenase Type IV (Gibco, catalog #17104019) in RPMI + GlutaMAX (Gibco) for 30 min at 37°C under agitation. Samples were filtered using a 70‐μm cell strainer and washed with RPMI + GlutaMAX (Gibco, catalog #61‐870‐036) supplemented with 10% FBS. After digestion, red blood cells were lysed using ACK Lysing Buffer (Gibco, catalog #A10492‐01) for 5 min at room temperature and washed using PBS. Blood was drawn in heparin, and RBC lysis was performed after antibody staining for flow cytometry.

### 2.10 Flow, Spectral Cytometry Staining Procedures

For flow and spectral cytometry, after tissue processing, cell suspensions were stained with Live/Dead Fixable Violet Stain Kit (catalog #L34955) in PBS for 15 min at room temperature. Cells were then washed and incubated with 1 μg/mL purified anti‐CD16/32 (BD Fc Block, clone 2.4G2, BD Biosciences, RRID:AB_394656, catalog #553142) for 10 min at room temperature; then surface staining was performed through an additional 30‐min incubation with surface marker antibodies. Cells were then fixed in PFA 4% for 20 min and then washed and resuspended in PBS containing 0.5% FBS, 0.01% sodium azide, and 2 mmol/L EDTA, and analyzed directly by flow cytometry. Data were acquired using a spectral cytometer (Cytek Aurora, Cytek Biosciences) for mouse samples or LSRFortessa X‐20 (BD Biosciences) for human LBA samples. Data were analyzed using FlowJo software v10.7.1 (Tree Star Inc., RRID:SCR_008520) for dot plot representation and cell quantification. The absolute cell number was recovered from the volume of cell suspension acquired by spectral cytometry.

The panel of antibodies used for mouse analysis included: anti‐CD45 (clone 30‐F11, BD Biosciences, RRID:AB_394003, catalog #567310), anti‐CD45 (clone 30‐F11, Biolegend, RRID:AB_2860600, catalog #103174), CD11b (clone M1/70, BD Biosciences, RRID:AB_2738276, catalog #563553), SiglecF (clone E50‐2440, BD Biosciences, RRID:AB_2739911, catalog #740158), CD177 (clone 241339F4, Biotechne, catalog #FAB8186G‐025), Ly6G (clone 1A8, BD Biosciences, RRID:AB_3684200, catalog #741813), Ly6C (clone HK1.4, Biolegend, RRID:AB_10897805, catalog #128028), CD43 (clone S7, BD Biosciences, RRID:AB_2916910, catalog #741067), I‐A/I‐E (clone M5/114.15.2, BD Biosciences, RRID:AB_2869739, catalog #566086), CD64 (clone X54‐5/7.1, Biolegend, RRID:AB_2563904, catalog #139314), anti‐CXCR4 (clone L276F12, Biolegend, RRID:AB_2562788, catalog #146511), anti‐PD‐L1 (clone 10F.9G2, Biolegend, RRID:AB_2563635, catalog #124321).

The panel of antibodies used for human LBA included: anti‐CD64 (clone 10.1, Biolegend, RRID:AB_2566236, catalog #305033), anti‐CD15 (clone W6D3, BD Biosciences, RRID:AB_2740635, catalog #741013), anti‐CD14 (clone M5E2, BD Biosciences, RRID:AB_2744285, catalog #564444), anti‐CD16 (clone 3G8, BD Biosciences, RRID:AB_10563252, catalog #329721), anti‐PD‐L1 (clone 29E.2A3, Biolegend, RRID:AB_2565763, catalog #560918).

### 2.11 In Situ Live Multiphoton Imaging of Lungs

Freshly explanted lungs of ACE2‐RGB‐mac mice were harvested and bathed in warm (37°C) oxygenated (100%) RPMI medium containing 10% FCS. Lung lobes were immobilized by applying gentle suction via gentle negative pressure through a thoracic imaging window to the exposed lung. The local temperature was monitored and maintained at 37°C during imaging. Oxygenated medium was renewed every 30 min. Real‐time videos were captured by imaging every 30 s for five consecutive image stacks with a z‐spacing of 3 μm (total thickness of 12 μm). For all images, the objective was a water immersion, ×20 apochromatic plane (numerical aperture = 1). For acquisition, the two‐photon laser scanning microscopy setup used was 7 MP (Carl Zeiss) coupled to a Ti:Sapphire crystal multiphoton laser (Chameleon Ultra, Coherent), which provides 140‐fs pulses of near‐infrared light, selectively tunable between 680 and 1050 nm and an optical parametric oscillator (OPO‐MPX, Coherent) selectively tunable between 1050 and 1600 nm. The excitation wavelengths were 820 nm for the nonlinear optical beam and 1070 nm for the optical parametric oscillator beam. The system included a set of external non‐descanned detectors in reflection with a combination of an LP‐600‐nm followed by LP‐462‐nm and LP‐500‐nm dichroic mirrors to split the light and collect the second harmonic generation signal and enhanced cyan fluorescent protein (ECFP) with a 480‐40‐nm emission filter, enhanced green fluorescent protein (EGFP) with a 525‐/50‐nm emission filter, mApple with a 624‐/40‐nm emission filter. Cell tracking was performed after 2D‐projections of z‐stack images using Imaris software (Bitplane, RRID:SCR_007370) to calculate mean speed, and arrest coefficient of neutrophils (defined as mApple^+^ cells localized in the lung parenchyma). Cells tracked for fewer than 90 s (three time points) were excluded. The arrest coefficient was defined as the proportion of time during which each cell's instantaneous velocity (calculated at every 30‐s interval) was < 2 μm/min. 3D images were performed on fixed lung lobes (PFA 4% over night).

### 2.12 Confocal Imaging

Lungs from ACE2‐RGB‐mac mice were harvested and fixed in 2% paraformaldehyde, 30% sucrose‐PBS overnight at 4°C before being embedded in OCT Tissue Freezing Medium (Microm Microtech, catalog #F/TFM‐C) and frozen at −80°C. Sectioning was completed on the HM550 Cryostat (Thermo Fisher Scientific) at −20°C; 5‐μm sections were collected on Superfrost Plus Microscope Slides (Thermo Fisher Scientific, catalog #22‐037‐246) and stored at −20°C until use. Slides were counterstained and mounted using VECTASHIELD Mounting Medium with 4,6‐diamidino‐2‐phenylindole (DAPI; Vector Laboratories, catalog #H‐1200). For some experiments, 5 μg of anti‐Ter119‐AF647 (clone TER‐119, Biolegend, RRID:AB_528961, catalog #116218) was injected intravenously to detect vascular leakage 2 min before harvesting the lungs.

Confocal images were acquired on a spinning disk microscope, Nikon Eclipse TI2E microscope (Nikon Corporation, Japan) linked with a Crest X‐Light V3 (CrestOptic, Italy) using Nikon NIS‐elements software. ECFP, EGFP, DAPI, and mApple signals were acquired using a combination of lasers from the CELESTA Light Engine (Lumencor, USA) and emission filters: laser 405 nm, EmBP 405/10 for DAPI; laser 440, EmBP460/25 for ECFP; laser 488, EmBP 510/20 for EGFP; laser 546, EmBP 603/30 for mApple. Images were analyzed using Imaris software (Bitplane). For image quantification, large and intermediate lung vessels were manually identified by the presence of surrounding elastin and counted across the entire lung section. The percentage of vessels filled with monocytes (RGB+ cells) and neutrophils (*R*+ cells) was determined for each section. Vessels were considered filled when RGB+ and *R*+ cells were in close contact, covering the majority of the luminal surface. Vessels were considered empty when no cells or only a few isolated cells were detected. The surfaces of R+/RGB+ cell aggregates were measured using ImageJ software, summed for each lung section, and normalized as a percentage of the total lung section area.

### 2.13 Mass Cytometry Imaging (Hyperion)

Raw data from mass cytometry imaging published in Guihot et al. (2022) were re‐analyzed for the indicated markers. All procedures and ethical statements are reported in the original publication. Briefly, two successive FFPE sections of 5 μm were used. The first section was stained with hematoxylin–eosin–saffron (HES) to allow the anatomopathologist to select the regions of interest (ROI). The second section was stained with an IMC panel containing the 30 metal‐conjugated antibodies and the cell intercalator (see Guihot et al. (2022)). Sections were deparaffinized with xylene and rehydrated through graded ethanol (100% to 70%) before transfer to TBS. Heat‐induced antigen retrieval was performed in a water bath at 95°C for 20 min in Tris/EDTA buffer (10 mM Tris, 1 mM EDTA, pH 9). Slides were cooled to room temperature and blocked with TBS + 3% BSA for 30 min. Slides were incubated with 100 μL of the antibody cocktail overnight at 4°C, washed three times with TBS, and labeled with Intercalator Ir (Fluidigm, 1:500 dilution) for 2 min at room temperature. After a brief rinse in water and air drying, IMC acquisition was performed on a Hyperion imaging system coupled to a Helios mass cytometer (Fluidigm Hyperion Imaging System) at a laser frequency of 200 Hz and laser power of 3 dB. For each ROI, 16‐bit single‐channel TIFF files were exported using MCD Viewer 1.0 (Fluidigm). Only channels/markers of interest were selected and overlaid to generate images using Imaris software. All images generated from these samples are displayed in the figure to illustrate inter‐individual variability.

### 2.14 Statistical Analysis

For multigroup analysis, each sample value was first tested for Gaussian distribution by the D'Agostino and Pearson omnibus normality test. As normality testing revealed the presence of at least one non‐Gaussian group across the datasets analyzed, all multigroup comparisons were conducted using the nonparametric Kruskal–Wallis test with Dunn's post hoc correction. For simple comparisons, Student's *t*‐test was used for parametric distributions and Mann–Whitney rank sum tests were used for non‐parametric distributions. The Spearman coefficient was calculated for correlation analysis. For survival analysis, the log‐rank Mantel–Cox test was performed. All these statistical analyzes were performed using GraphPad Prism 7 software (RRID:SCR_002798). The sample sizes and statistical tests are indicated in each figure legend. Data are expressed as mean ± SEM unless otherwise specified. Statistical significance is defined as *p* < 0.05. *p < 0.05; **p < 0.01; ***p < 0.001; ****p < 0.0001. Multivariable logistic regressions with interaction terms were fitted using the *glm* function in R software and ROC analyzes were performed using the pROC package. Both analyzes were fitted on the overall study population, including patients from two age‐defined cohorts. Although patients originated from distinct age‐based cohorts, adjustment for age and sensitivity analyzes were performed to account for population heterogeneity. Disease severity was modeled as a binary outcome (non‐severe/non‐critical vs. critical). Neutrophil‐to‐monocyte ratio (NMR), neutrophil‐to‐lymphocyte ratio (NLR), age, and sex were included as covariates, with continuous variables standardized using *z*‐scores. Interaction terms were introduced to assess effect modification by age and sex. Results are reported as adjusted odds ratios with 95% confidence intervals. Multicollinearity was assessed using variance inflation factors and remained < 2 for all variables, indicating no collinearity. Analyzes were conducted on complete cases, and statistical significance was defined as *p* < 0.05. Wald tests were performed for multivariable logistic regression, and DeLong tests were performed to assess AUC differences.

## 3 Results

### 3.1 COVID‐19 Severity Is Associated With an Increased Neutrophil‐To‐Monocyte Ratio (NMR) in Mice

**FIGURE 1:**
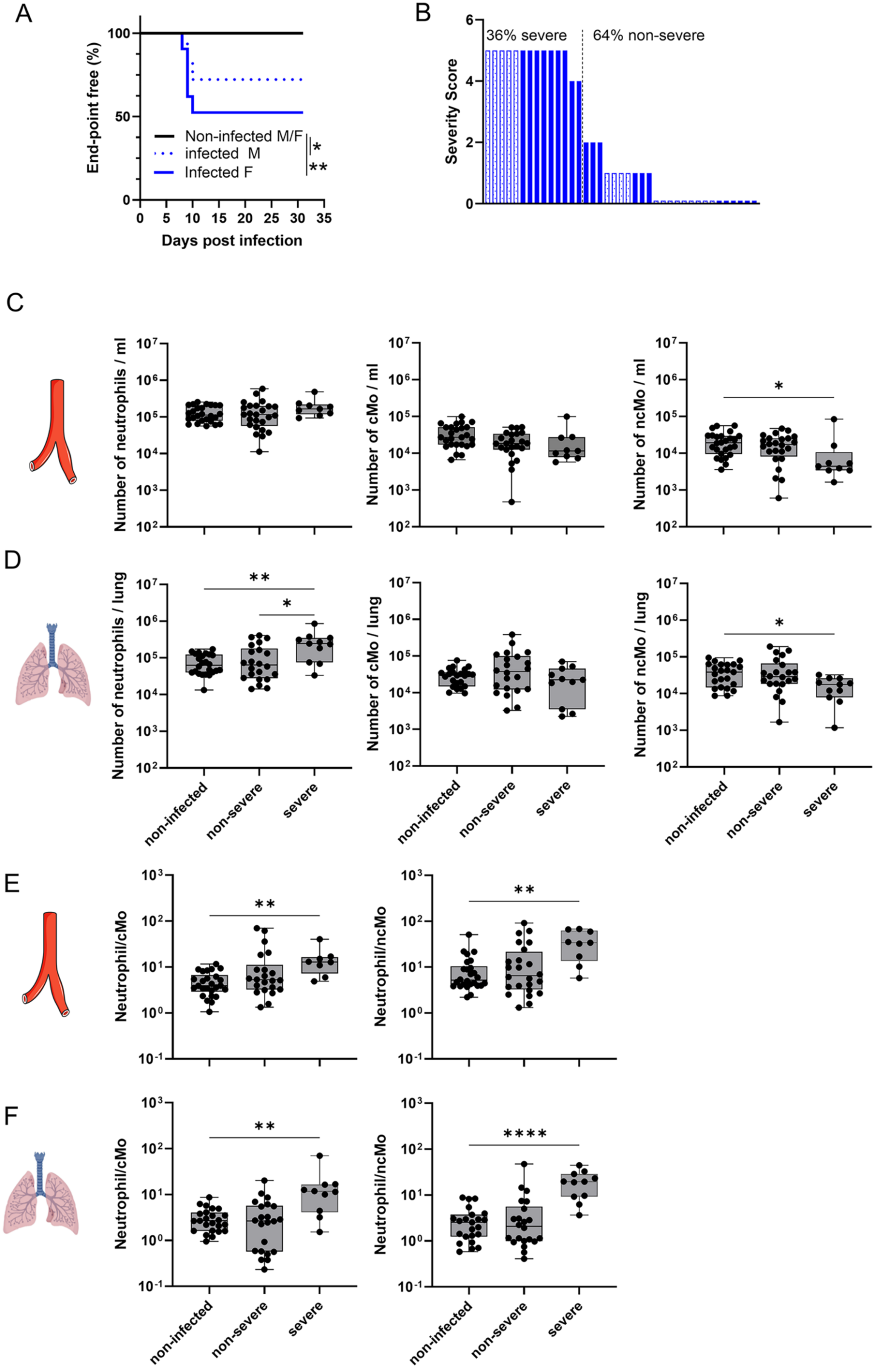
COVID‐19 severity is associated with an increased neutrophil‐to‐monocyte ratio (NMR) in mice. (A) Kaplan–Meier survival curves after SARS‐CoV‐2 intranasal inoculation (0.31 PFU) of males (*n* = 18), females (*n* = 21) and non‐infected males and females pooled (*n* = 13). End‐point was considered when severity scoring reached 5. Log‐rank Mantel–Cox test was performed between infected and non‐infected mice. M vs. F difference was not significant. Data are pooled from two independent experiments. (B) Cascade bar graph shows the maximum severity score reached for each mouse before end‐point or clinical remission. (C, D) Whisker plots show the number of indicated cells per mL of blood (C) and per total lung (D) at day 7 after infection according to the clinical status of the mice. (E) Whisker plots show the NMR in the blood (E) and the lungs (F) at day 7 after infection according to the clinical status of the mice. For all panels, severe clinical status was considered for a score ≥ 3, and non‐severe ≤ 2. Each dot represents one mouse. Data are pooled from at least three independent experiments. Kruskal‐Wallis with Dunn's multiple comparison tests were performed. *p < 0.05; **p < 0.01; ****p < 0.0001. See also Figure S1.

To investigate the relationship between COVID‐19 severity and the myeloid response, K18‐ACE2 transgenic mice were infected with a SARS‐CoV‐2 strain isolated from a hospitalized patient in 2020 at a standardized dose designed to result in less than 50% mortality in mice younger than 6 months old (Figure 1A). Clinical status was monitored longitudinally following infection. A severity score above 2 was considered clinically severe, whereas a score of 2 or below was classified as non‐severe (Figures 1B and S1A). No significant difference in clinical severity was observed between males and females; therefore, immune phenotyping was next performed on females exclusively. Viral load in lung tissues was assessed by RT‐qPCR in three independent experiments across the whole study and was detectable in 87.5% of tested mice (Figure S1B). Although viral load varied substantially between mice, it was not associated with clinical severity. Neutrophils were defined as CD45+ CD11b+CD177+Ly6G+ in both blood and lungs, classical monocytes (cMo) were defined as CD45+CD11b+Ly6C+CD64+/−, and non‐classical monocytes (ncMo) as CD45+CD11b+CD43+ CD64+/−, according to the gating strategy (Figure S1B,C). Regardless of clinical severity, the absolute numbers of neutrophils, classical monocytes (cMo) and non‐classical monocytes (ncMo) in the blood were not significantly altered at 4, 7, and 17 days post‐infection (Figure S1D). In the lungs, some significant variations were noticed, despite substantial inter‐individual variability, particularly in the absolute numbers of neutrophils, cMo and ncMo at day 7 (Figure S1E). When stratified by clinical severity, the absolute number of neutrophils was significantly increased in the lungs of mice with severe disease compared with both non‐infected mice and mice with non‐severe infection. This increase was not observed in the blood (Figure 1C,D, neutrophil panels). In contrast, the number of cMo showed a slight but not significant decrease, while ncMo numbers were reduced in both blood and lungs of mice with severe disease (Figure 1C,D, monocyte panels). Consistent with this observation, the neutrophil‐to‐monocyte ratio (NMR) was markedly increased in both blood (Figure 1E) and lungs (Figure 1F) of mice with severe disease. These findings suggest that an imbalance between these myeloid subsets accompanies disease progression.

### 3.2 Monocytes and Neutrophils Aggregate in the Lung Vasculature During SARS‐CoV‐2 Infection

**FIGURE 2:**
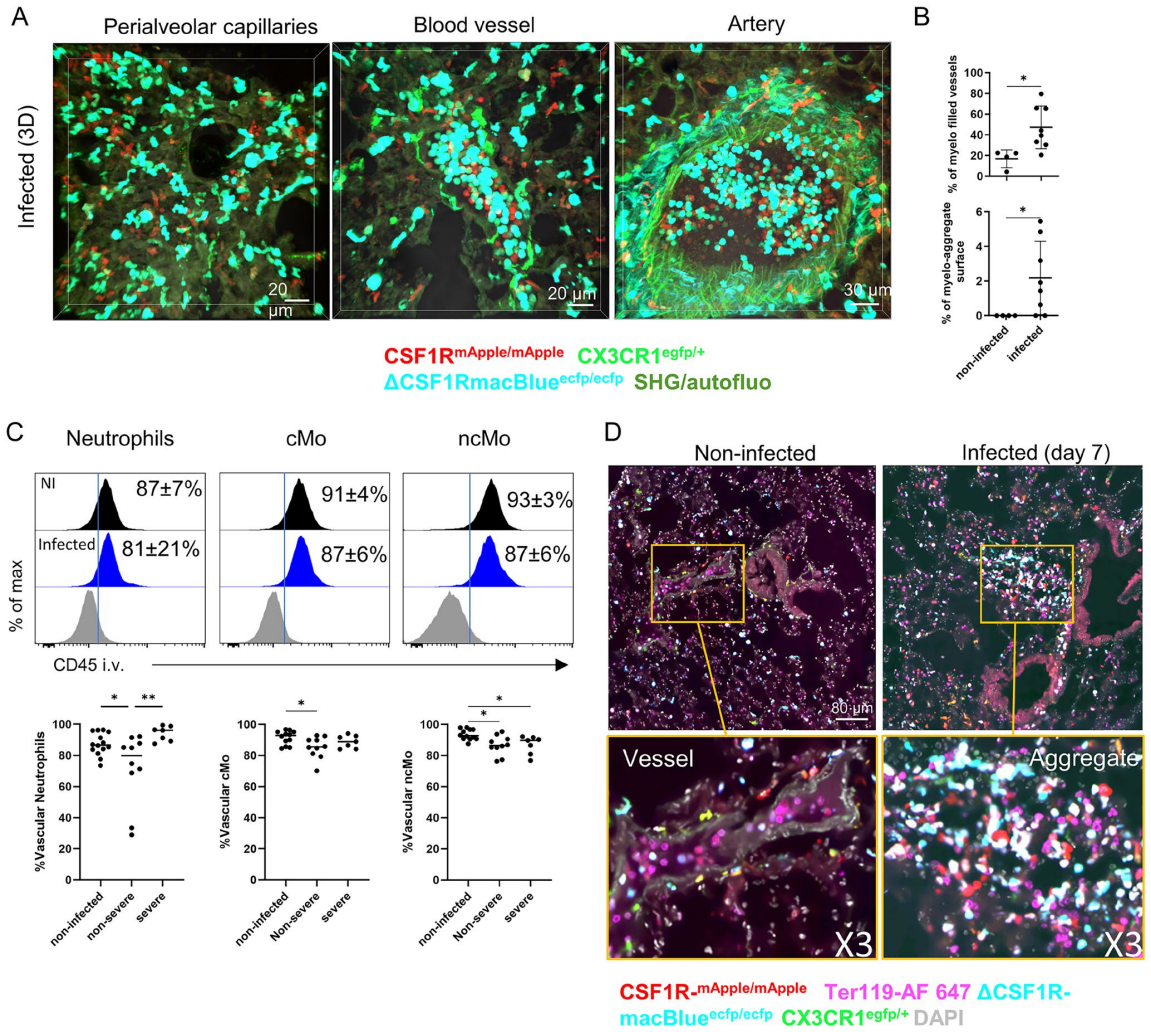
Monocytes and neutrophils aggregate in the lung vasculature during SARS‐CoV‐2 infection. (A) 3D Two‐photon laser scanning microscopy (TPLSM) images of ACE2‐RGB‐mac transgenic mouse fixed lung lobes, showing monocyte and neutrophil vascular aggregation 6 days after SARS‐CoV‐2 inoculation. According to Figure S2A, monocytes and interstitial Mo‐derived macrophages express mApple, EGFP, and ECFP (RGB+ signature), neutrophils express mApple (*R*+ signature). Collagen fibers and elastin around blood vessels and arteries are detected by second harmonic generation (SHG). Epithelial structures are detected by autofluorescence. (B) Image quantification of the percentage of lung vessels filled with R+/RGB+ cells (as represented in A, D and Figure S2C) and fraction of the lung surface covered with R+/RGB+ cell aggregates (as represented in A, D and Figure S2B,C). Each dot represents one lung section from one mouse. Mann–Whitney tests were performed. (C) Representative histogram plots show blood tissue partitioning of neutrophils, cMo, and ncMo by CD45 intravascular staining (using intravenous injection of anti‐CD45‐PerCP‐Cy5.5 before tissue harvest) 7 days after infection. Percent ±SD of CD45i.v. positive cells are indicated based on negative control staining (gray histogram). Scatter plots show the % of intravascular resident cells (CD45i.v.+) according to the clinical status 7 days after infection. Each dot represents one mouse, data are pooled from two independent experiments. Kruskal‐Wallis with Dunn's multiple comparison tests were performed. *p < 0.05; **p < 0.01. (D) Confocal images of lung cryosections from infected (day 6) or non‐infected ACE2‐RGB‐mac transgenic mouse show the co‐aggregation of neutrophils (*R*+ cells) and monocytes (RGB+ cells) with Ter119+ erythrocytes. Anti‐Ter119‐AF647 was injected intravenously 2 min before harvesting the lungs. See also Figure S2.

To further investigate the impact of SARS‐CoV‐2 infection on the myeloid response, we generated the ACE2 RGB‐Mac mouse. The RGB‐mac model combines multiple fluorescent reporters enabling in situ visualization of all myeloid subsets in the lung (Petit et al. 2024) (Figure S2A). The relative expression of the red (mApple), green (GFP), and blue (ECFP) fluorescence allowed the identification of neutrophils which express only the red fluorescence (R+), and their distinction from both cMo and ncMo, which express combined red, green, and blue fluorescence (RGB+) (Figure S2A). Histological analysis of infected lungs revealed no global accumulation of *R*+ and RGB+ cells compared to non‐infected lungs, except in localized regions where dense cellular aggregates were observed, suggestive of parenchymal lesions (Figures 2A,B and S2B,C). These aggregates consisted of both *R* ^+^ and RGB^+^ cells, corresponding to neutrophils and monocytes, respectively. They were detected within the alveolar space but predominantly in larger vessels, suggesting that neutrophils and monocytes primarily reside in the lung vasculature during infection (Figures 2A and S2C), consistent with their established association with thrombotic processes (Middleton et al. 2020). Blood/tissue partitioning, as described (Petit et al. 2024), was performed using intravascular staining with anti‐CD45‐PerCP‐Cy5.5 (CD45i.v.). Seven days post‐infection, more than 80% of neutrophils and over 87% of both cMo and ncMo were labeled with the fluorescent antibody, similar to non‐infected mice, confirming their predominant localization within the vascular compartment of lung capillaries and larger vessels (Figure 2C). Severe clinical symptoms were associated with a higher proportion of neutrophils residing in the vascular compartment compared with non‐severe cases whereas this trend was less pronounced for monocytes (Figure 2C). Finally, in vivo labeling of erythrocytes with an anti‐Ter119 antibody, administered intravenously 2 min before harvesting the lungs, demonstrated that neutrophil and monocyte aggregates contained Ter 119+ cells confined to the lung microvasculature, with no evidence of dense accumulation indicative of vascular leakage (Figure 2D).

Together, these results indicate that COVID‐19 severity is associated with the accumulation of neutrophils and their aggregation with monocytes within large vessels and microcapillaries of the lungs.

### 3.3 Ly6G‐Dependent Trapping of Neutrophils in the Lung Microvasculature Is Associated With COVID‐19 Severity

**FIGURE 3:**
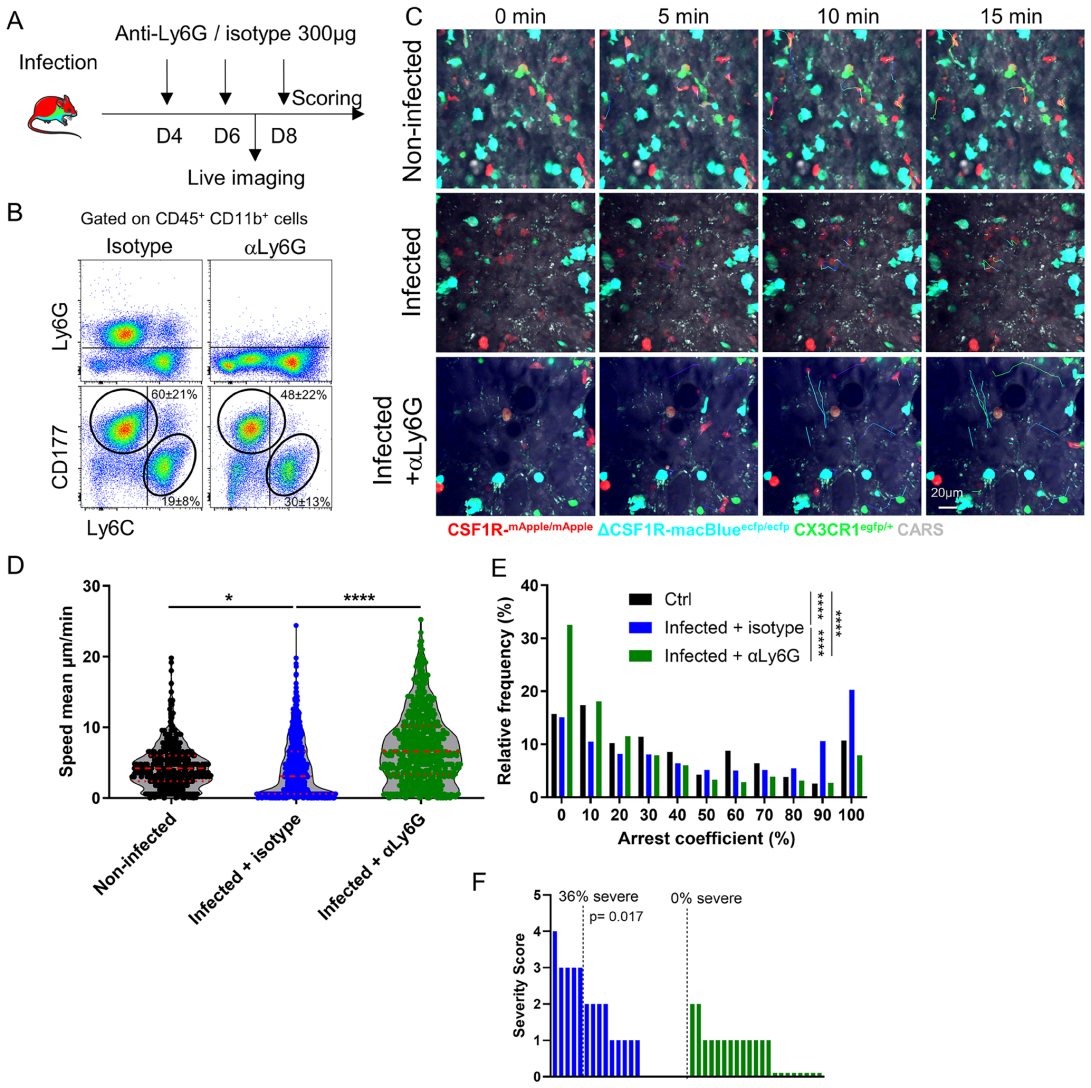
Ly6G‐dependent trapping of neutrophils in the lung microvasculature is associated with COVID‐19 severity. (A) Protocol of anti‐Ly6G treatment after SARS‐CoV‐2 inoculation. Live imaging of explanted lungs was performed at day 6 on ACE2‐RGB‐Mac mice, and clinical scoring was performed on ACE2 mice. (B) Representative dot plots showing the neutralization of Ly6G expression on lung neutrophils after anti‐Ly6G or isotype control (upper plots). Percent±SD of CD177+ Ly6C^low^ neutrophils and CD177‐ Ly6C+ monocytes are indicated (lower plots) (*n* = 4). (C) Representative TPLSM image sequence showing the migratory behavior of neutrophils. Colored lines indicate representative track paths over time of neutrophils (*R*+ cells in red) in different conditions 6 days after infection. (D) Violin plots show the neutrophil mean velocity for each condition. Means and quartiles are indicated (dashed red lines). (E) Relative distribution of neutrophil arrest coefficients for each condition. For (D, E), data are pooled from *n* = 6, 7, and 6 mice, for non‐infected, infected + isotype, and infected + anti‐Ly6G groups, respectively. Neutrophil motility was similar with or without isotype control treatment in infected mice, so the data were pooled for this condition. Kruskal‐Wallis with Dunn's multiple comparison tests were performed. **p < 0.01; ****p < 0.0001. (F) Cascade bar graphs show the maximum severity score reached for each mouse before end‐point or clinical remission after anti‐Ly6G (*n* = 21) or isotype control (*n* = 14) treatments. Dashed lines indicate the threshold of severe scoring ≥ 3. Fisher's exact test was performed.

To further investigate the role of neutrophils following SARS‐CoV‐2 infection, we compared the clinical outcomes in mice treated three times with anti‐Ly6G or isotype control between day 4 and day 8 after virus inoculation (Figure 3A). Anti‐Ly6G treatment has previously been shown not to fully deplete neutrophils but instead to induce rapid medullary release of neutrophils lacking Ly6G expression (Pollenus et al. 2019; Boivin et al. 2020). Consistent with these studies, the anti‐Ly6G clone 1A8 used here did not completely deplete neutrophils, with their frequency decreasing from 80% to 48%, corresponding to an approximate 40% reduction. However, membrane Ly6G was no longer detectable, whereas Ly6C expression remained unchanged on both neutrophils and monocytes, likely reflecting epitope masking or antibody‐mediated neutralization (Figure 3B). We next performed two‐photon live imaging on freshly explanted lung lobes from ACE2‐RGB‐Mac mice to compare neutrophil migratory behavior across conditions (Figure 3C and Videos S1–S3). Due to the difficulty in discriminating monocytes from inflammatory macrophages (RGB+ cells) accumulating at the lung surface, our analysis focused exclusively on neutrophil dynamics (*R*+ cells). SARS‐CoV‐2 infection resulted in decreased mean velocity (Figure 3D) and increased arrest coefficient (Figure 3E and Video S2) of neutrophils compared with non‐infected mice (Video S1), consistent with their enhanced accumulation within the vasculature. In the presence of anti‐Ly6G, neutrophils displayed increased mean velocity and a marked reduction in arrest coefficient (Figure 3D,E and Video S3), suggesting a reduced microvascular obstruction of the lung, as previously reported in the brain (El Amki et al. 2020). Importantly, anti‐Ly6G treatment significantly reduced clinical severity (Figure 3F). Notably, the severity score represents an integrated measure of clinical manifestations, including central nervous system‐driven signs such as tremor, hypothermia, and reduced mobility; therefore, the observed therapeutic effect cannot be attributed solely to pulmonary pathology.

Taken together, these results suggest that reduced neutrophil motility and their trapping within the lung microvasculature is detrimental, supporting a pathogenic contribution to severe clinical outcome during SARS‐CoV‐2 infection.

### 3.4 COVID‐19 Severity Is Associated With a Defect in Classical Monocyte Activation in the Lung

**FIGURE 4:**
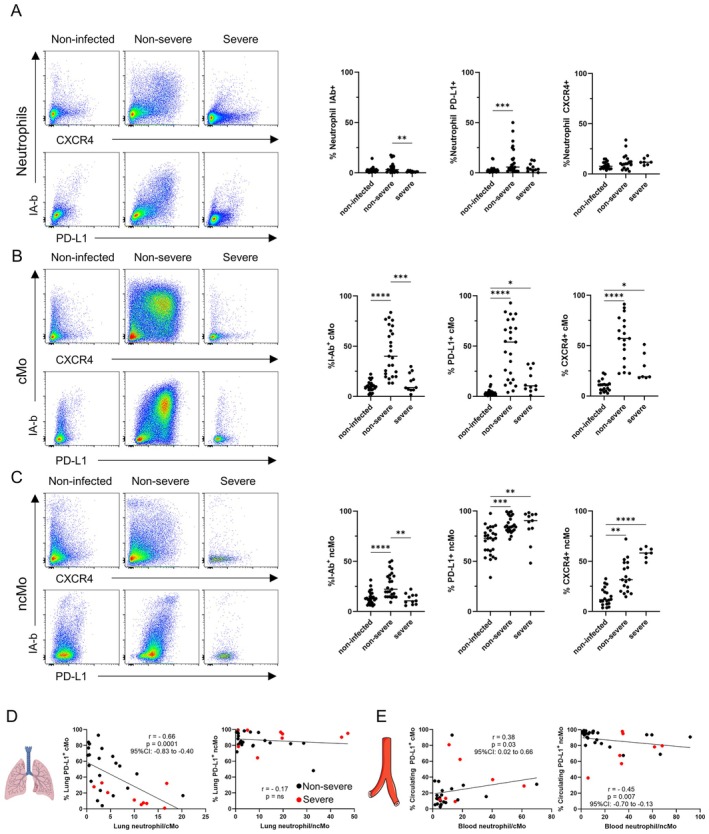
COVID‐19 severity is associated with a defect in classical monocyte activation in the lung Representative flow cytometry dot plots and quantification of PD‐L1, I‐Ab, and CXCR4 expression on lung (A) neutrophils, (B) classical monocytes cMo, (C) non‐classical monocytes ncMo, according to the clinical status, 7 days after SARS‐CoV‐2 inoculation. Cells were identified based on the gating strategy in Figure S1. Kruskal‐Wallis with Dunn's multiple comparison tests were performed. *p < 0.05; **p < 0.01; ***p < 0.001; ****p < 0.0001. Correlation analysis of the % of PD‐L1+ cMo and ncMo subsets as a function of the NMR in (D) the lungs, (E) the blood. The Spearman coefficient of correlation *r* and 95% confidence interval (CI) are indicated along with the *p*‐value. Straight lines represent linear regression. See also Figure S3.

We next investigated the activation states of circulating and lung associated neutrophil and monocyte subsets in relation to the severity of the disease. Neutrophils recovered from the lungs barely upregulated I‐Ab, 7 days after infection (5% ± 5% in non‐severe, 1.3% ± 0.7% in severe) compared with 3% ± 3% in non‐infected mice (Figure 4A). In the blood, the percentage of I‐Ab+ neutrophils was significantly higher in non‐severe (10% ± 9%) than in mice with severe symptoms (1.5% ± 1%) (Figure S3A). PD‐L1 expression on both lung‐associated and circulating neutrophils was heterogeneous among mice. A significant upregulation was observed in non‐severe mice compared with non‐infected controls, whereas this increase was absent in mice with severe symptoms (Figures 4A and S3A). The percentage of CXCR4+ neutrophils was lower in the lung than in circulation (8% ± 3% vs. 16% ± 14% in non‐infected mice) and was not significantly altered upon viral infection, likely due to the high inter‐individual variability of responses, regardless of COVID‐19 severity (Figures 4A and S3A). In lung‐associated cMo, the percentages of I‐Ab+, PD‐L1+ and CXCR4+ cells were higher than in neutrophils and were significantly increased in non‐severe disease compared with both severe and non‐infected conditions, although with a substantial heterogeneity (Figure 4B). In contrast, circulating cMo showed weaker or non‐significant upregulation of these markers, with no association with disease severity (Figure S3B), suggesting a tissue‐dependent activation of cMo. This association was specific to cMo. In lung associated ncMo, I‐Ab upregulation was also higher in non‐severe disease, but PD‐L1 and CXCR4 expression increased with COVID‐19 severity (Figure 4C). Conversely, PD‐L1 expression on circulating ncMo was elevated only in non‐severe compared with both non‐infected mice and mice with severe symptoms (Figure S3C), reflecting an almost opposite activation profile between lung‐resident and circulating cMo and ncMo. The proportion of infiltrating cells among PD‐L1+ and PD‐L1‐ monocytes and neutrophils, using blood/tissue partitioning, revealed that PD‐L1+ cMo were more prone to infiltrate the lungs compared to other subsets. This suggests that PD‐L1+ cMo infiltration in the lung parenchyma is associated with a positive clinical outcome (Figure S3D). To assess the functional relevance of PD‐L1, young SARS‐CoV‐2–infected mice were treated with an anti–PD‐L1 antibody every 2 days from day 4 to day 8 post‐infection. Although this treatment markedly reduced detectable PD‐L1 expression on circulating monocytes and neutrophils (Figure S3E), it did not affect clinical outcomes (Figure S3F) or the numbers of circulating neutrophils, cMo, or ncMo (Figure S3G). Therefore, this approach did not allow us to determine the in vivo role of PD‐L1+ cMo in this setting. Finally, a significant inverse correlation was observed between the frequency of PD‐L1+ cMo and the neutrophil‐to‐cMo ratio in the lung, whereas no such relationship was detected in circulating cells (Figure 4D). This correlation was not observed for ncMo.

Overall, these findings indicate that disease severity is associated with subset‐ and tissue‐specific monocyte dysregulated responses rather than a uniform defect across the entire monocyte compartment.

### 3.5 Age‐Related COVID‐19 Severity Is Associated With an Accumulation of Neutrophils and a Reduced PD‐L1 Upregulation by Classical Monocytes in the Lungs

**FIGURE 5:**
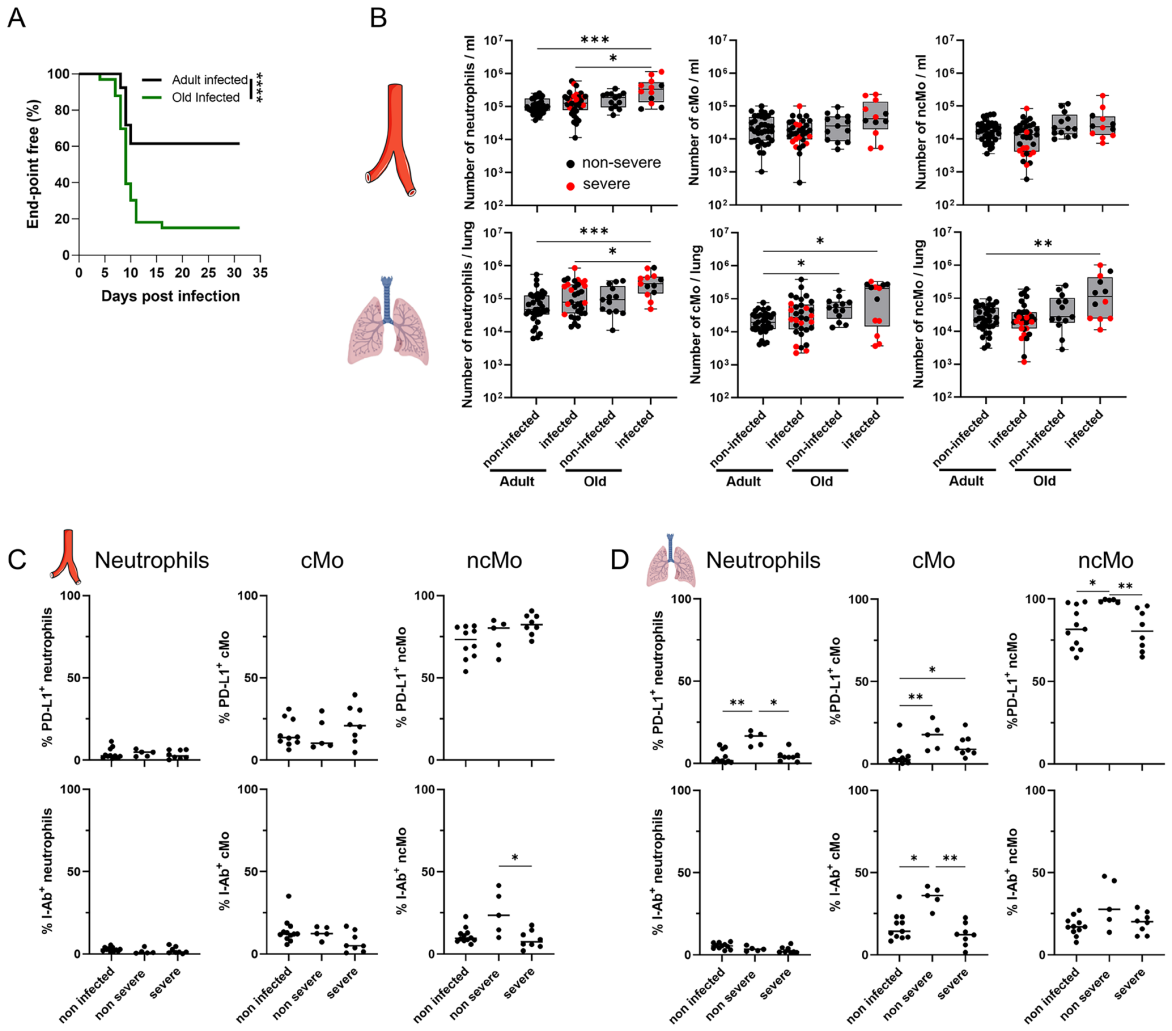
Age‐related COVID‐19 severity is associated with an accumulation of neutrophils and a reduced PD‐L1 upregulation by classical monocytes in the lungs. (A) Kaplan–Meier survival curves after SARS‐CoV‐2 intranasal inoculation (0.31 PFU) of adult (4 ± 1 months old) males (*n* = 11), females (*n* = 28) and old (24 ± 2 months old) males (*n* = 11), females (*n* = 22). End‐point was considered when severity scoring reached 5. Log‐rank Mantel–Cox test was performed between adult and old infected mice. No difference between M and F was observed. Data are pooled from three independent experiments. (B) Whisker plots show the number of indicated cells per mL of blood (upper panels) and per total lung (lower panels), 7 days after SARS‐CoV‐2 inoculation. Cells were identified based on the gating strategy in Figure S1. Each dot represents one mouse. Red dots indicate mice exhibiting severe symptoms (score ≥ 3). Data are pooled from two independent experiments pairing adult and old female mice. Data for adult mice are also included in Figure 1. Kruskal‐Wallis with Dunn's multiple comparison tests were performed. *p < 0.05; **p < 0.01; ***p < 0.001; ****p < 0.0001. (C, D) Quantification of PD‐L1 and I‐Ab expression on neutrophils, classical monocytes (cMo) and non‐classical monocytes (ncMo) in the blood (C) and lungs (D) of old mice, according to the clinical status, 7 days after SARS‐CoV‐2 inoculation. For all panels, data are pooled from two independent experiments. Kruskal‐Wallis with Dunn's multiple comparison tests were performed. *p < 0.05; **p < 0.01; ****p < 0.0001. See also Figure S4.

We next investigated the relation between neutrophil accumulation and PD‐L1 expression on cMo in the context of age‐related severity of SARS‐CoV‐2 infection. As observed in humans, the clinical outcome of aged mice (24 ± 2 months old) was severely worse than that of younger adult mice (4 ± 1 months old) following SARS‐CoV‐2 infection (Figure 5A). Seven days post‐infection, the number of circulating neutrophils and lung neutrophils was significantly higher in aged mice compared with younger adults, irrespective of disease severity (Figure 5B). The numbers of cMo and ncMo also tended to be higher in aged infected mice, as well as in non‐infected controls, probably reflecting a basal level of inflammaging (Figure 5B). Consequently, the neutrophil‐to‐monocyte ratio did not differ significantly between aged and younger mice overall, although it remained elevated in aged mice with severe symptoms (Figure S4). Finally, aged mice exhibited a reduced capacity for both circulating (Figure 5C) and lung‐associated (Figure 5D) cMo and ncMo to upregulate PD‐L1, even among the few old mice with less severe clinical symptoms. Interestingly, PD‐L1 upregulation on cMo was associated with the acquisition of CD64 expression, linking monocyte activation to their progressive differentiation into macrophages (Figure S4B,C). In young mice with non‐severe symptoms, macrophage differentiation occurred within the vascular compartment (CD45i.v. + cells) and was enriched among infiltrating cMo (CD45i.v.−cells). In contrast, in young mice with severe symptoms, cMo activation and differentiation were markedly impaired (Figure S4B), a phenomenon also observed in aged mice exhibiting non‐severe symptoms (Figure S4C).

These findings suggest that age‐dependent dysregulation of the myeloid response contributes to increased susceptibility to severe COVID‐19.

### 3.6 Human Aging Is Associated With Elevated Blood NMR and Reduced PD‐L1 Expression on Lung Monocytes During SARS‐CoV‐2 Infection

**FIGURE 6:**
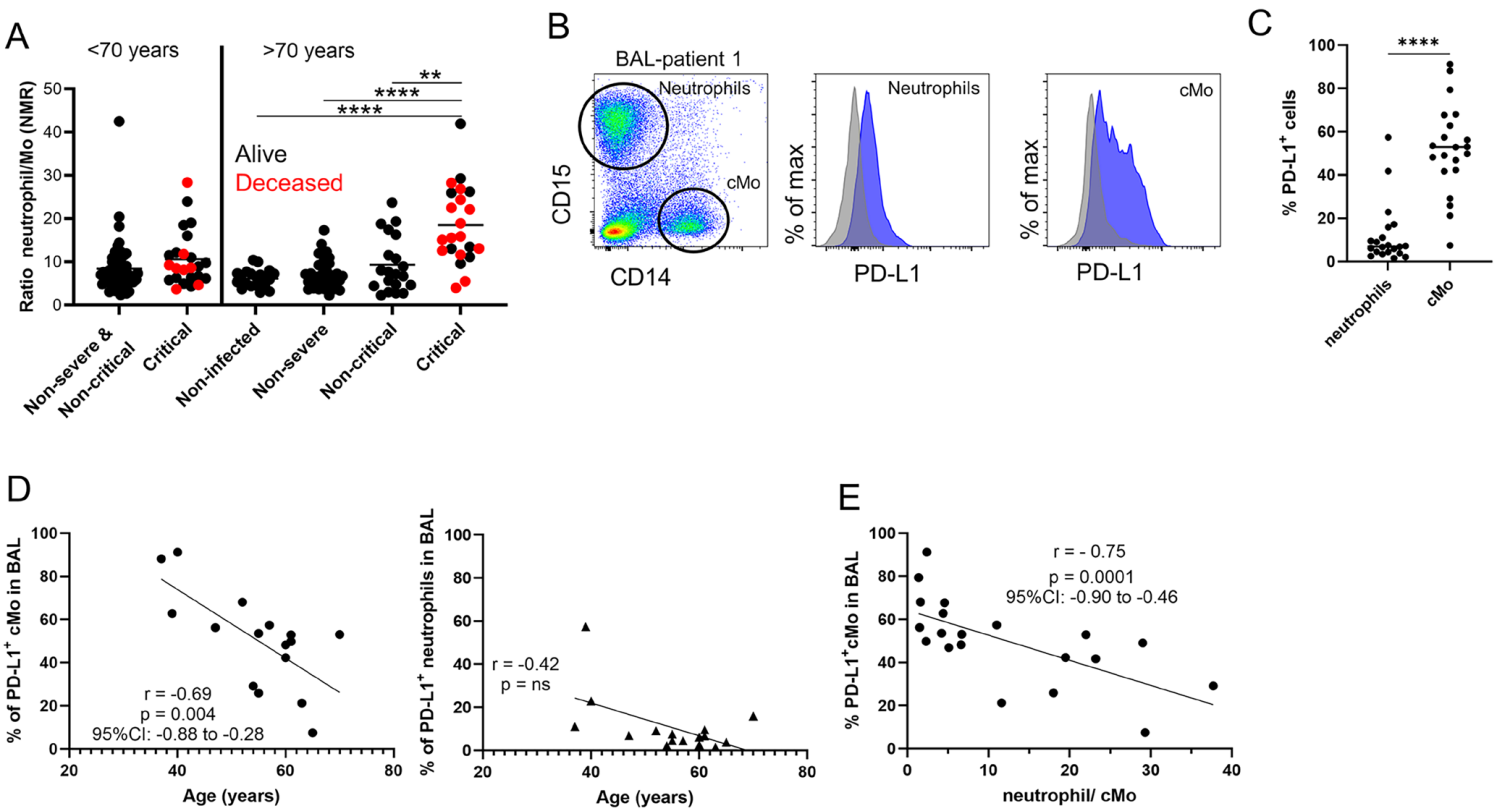
Human aging is associated with elevated blood NMR and reduced PD‐L1 expression on lung monocytes during SARS‐CoV‐2 infection. (A) Scatter plots show the NMR, according to clinical severity and age of the patients. Each dot represents one patient. Red dots indicate deceased patients in the following days. Data are extracted from the indicated publications. For patients < 70 years old, Mann–Whitney tests were performed. For patients > 70 years old, Kruskal‐Wallis with Dunn's multiple comparison tests were performed. **p < 0.01; ***p < 0.001; ****p < 0.0001. (B) Representative flow cytometry plots of bronchoalveolar lavage (BAL) of SARS‐CoV‐2 infected patients showing the gating of CD15+ neutrophils (confirmed with CD16low/high expression) and CD14+ classical monocytes (cMo) (confirmed with CD64+ CD16‐ expression), and PD‐L1 expression. Gray histograms represent FMO control staining. (C) Quantification of PD‐L1 expression on neutrophils and cMo recovered from BAL. Each plot represents one patient. (D, E) Correlation analysis of the % of PD‐L1+ cMo and neutrophils from the BAL as a function of the age of the patients (D) or as a function of the neutrophil‐to‐cMo ratio (E). The Spearman coefficient of correlation *r* and 95% confidence interval (CI), are indicated along with the *p*‐value. Straight lines represent the linear regression. ns, non‐significant. See also Figures S5 and S6.

We next assessed whether these observations were transposable to human COVID‐19. We analyzed two previously published cohorts of patients including: patients younger than 70 years from Kilercik et al. (2021) and elderly patients older than 70 years from Poisson et al. (2024). Absolute counts of circulating neutrophils and monocytes were compared (Figure S5A) to calculate the NMR according to disease severity (Figure 6A). In both cohorts, circulating neutrophil counts were higher in patients with critical clinical status compared to those with non‐critical or non‐severe symptoms (Figure S5A). In contrast, monocyte counts were not affected by the clinical status in either age group (Figure S5A). Notably, NMR was significantly elevated in elderly patients with critical condition but not in the younger cohort (Figure 6A). The neutrophil‐to‐lymphocyte ratio (NLR) was increased in patients with critical condition in both cohorts (Figure S5B). Multivariate logistic regression analyzes including patients from the two age‐defined cohorts showed that NLR was strongly associated with disease severity independently of age (OR = 8.82, *p* = 0.007) (Figure S5C). In contrast, NMR did not show a significant main effect (OR = 1.65, *p* = 0.24), but exhibited a significant interaction with age (OR = 1.80, *p* = 0.043), indicating that its association with disease severity increases with advancing age. The interaction between NLR and age showed a non‐significant trend (*p* = 0.07) and no significant effect modification by sex was observed for either biomarker. Variance inflation factors were all below 2, indicating no collinearity (Figure S5C). These results indicate that NMR is not simply a surrogate marker of NLR, but rather a complementary biomarker whose prognostic relevance appears age‐dependent. As the multivariable analysis suggested an interaction between NMR and age, receiver operating characteristic (ROC) curves were generated to assess the predictive value of NMR and NLR through their respective interactions with age (NLR × Age, NMR × Age) as well as their combined interaction (NLR × NMR × Age). The area under the curve (AUC) for NMR × Age was significantly lower than the AUC for NLR × Age (0.74 vs. 0.80, respectively) (Figure S5C), confirming the potency of NMR as a surrogate marker of COVID‐19 severity along with NLR but with no added value compared to NLR, as depicted by the AUC of the combined (NLR × NMR × Age) model (0.78).

We next investigated the effect of SARS‐CoV‐2 infection in human lungs. Mass cytometry imaging of lung sections from three deceased SARS‐CoV‐2 infected patients (Figure S6A) and two control patients with cardiovascular diseases (Figure S6B) revealed co‐aggregation of monocytes (CD14+) and neutrophils (MPO+) in SARS‐CoV‐2 infected lungs (Figure S6). To assess the expression of PD‐L1 on lung monocytes, we analyzed the flow cytometry data from bronchoalveolar lavages (BAL) from a previously published cohort (Guihot et al. 2022). Among classical monocytes defined as CD14+CD16‐CD64+, 52% ± 21% expressed PD‐L1, whereas only 12% ± 14% of neutrophils defined as CD15+CD16+/− were PD‐L1 (Figure 6B,C). Consistent with our previous observation in mice, PD‐L1 expression on monocytes, but not on neutrophils, inversely correlated with patient age (Figure 6D), indicating a progressive decline of PD‐L1 upregulation with aging. It is noteworthy that in this cohort, patients were all on mechanical ventilation; hence, severity stratification was not possible, reflecting an age and not severity‐dependent loss of PD‐L1 expression. Similarly, the frequency of PD‐L1+ cMo in BAL inversely correlated with NMR, as observed in the mouse model (Figure 6E).

We concluded that, in humans, increased blood and lung NMR and reduced PD‐L1 expression on BAL monocytes reflect age‐dependent features associated with severe SARS‐CoV‐2 infection, highlighting an age‐related dysregulation of the myeloid response.

## 4 Discussion

By integrating mouse models, intravital lung imaging, and human patient cohorts, we showed that SARS‐CoV‐2 infection is associated with excessive neutrophil accumulation and vascular arrest in the lungs, whereas protection correlates with the presence of PD‐L1+ classical monocytes infiltrating lung tissue. These opposing inflammatory and immunoregulatory myeloid signatures appear closely linked to COVID‐19 severity. Aging profoundly disrupts this balance, favoring neutrophil‐driven pathology while impairing the induction of PD‐L1 on lung monocytes, thereby predisposing elderly individuals to severe disease.

In this study, we used an unprecedented low‐dose viral inoculum based on in vitro titration. For each virus preparation, we established a dose–response calibration by progressively decreasing the viral inoculum to identify an experimental window capable of generating heterogeneous disease outcomes while avoiding uniformly lethal infection. The dose of approximately 0.31 PFU per mouse was selected because it consistently produced an intermediate phenotype (close to 50% survival). In vivo titration confirmed detectable viral copies in the lungs in 87.5% of tested mice, and we did not observe a correlation between viral load and disease severity, arguing against a dose‐dependent artifact as the primary driver of outcome heterogeneity. Because infected mice were bred in groups, cross‐contamination was most likely to occur, which may have contributed to effective viral exposure despite the low inoculum and complicates the direct relationship between the in vitro dose and the infection rate in the colony. This low dose infection may introduce stochastic elements in the initiation of infection but nevertheless reflects the principle of infection at a population scale and revealed a clear susceptibility of aged mice to higher severity. Interestingly, even in non‐severe infected mice, a clear monocyte activation profile characterized by upregulation of PD‐L1, CXCR4, I‐Ab, and CD64 expression was still observed in the blood and the lung, further supporting effective infection even in the absence of clinical symptoms.

Our findings reinforce the central pathogenic role of myeloid cell dysfunction in severe COVID‐19 (Schulte‐Schrepping et al. 2020; Silvin et al. 2020). Using live imaging, we provided new mechanistic insight into the impact of neutrophil accumulation in the lung microvasculature, showing that SARS‐CoV‐2 infection induces a marked reduction in neutrophil motility and an increased arrest coefficient, consistent with microvascular plugging rather than widespread parenchymal infiltration. This phenotype is exacerbated in severe disease and functionally linked to outcome, as partial interference with Ly6G‐dependent neutrophil arrest significantly reduced clinical severity in mice. These observations align with extensive literature implicating neutrophil‐mediated immunothrombosis (Combadière et al. 2021), endothelial injury, and NET formation as major drivers of acute respiratory distress syndrome (ARDS) and multiorgan failure in COVID‐19 (Barnes et al. 2020; Hazeldine and Lord 2021). Nevertheless, the persistence of neutrophils within the vascular compartment without evidence of major vascular leakage or NET formation further supports a model in which intravascular neutrophil aggregation and flow obstruction are key contributors to lung dysfunction, consistent with prior observations in other organs, including the brain (El Amki et al. 2020).

It is likely that similar patterns in neutrophil and monocyte accumulation occur in other tissues and that lung associated immune mechanisms represent one component of disease heterogeneity. For instance, SARS‐CoV‐2 neurotropism has been reported (Huang et al. 2026; Meinhardt et al. 2021). The lowest intranasal viral dose of 10^2^ PFU leading to neuroinvasion with limited neuroinflammation was reported in a recent, yet not peer‐reviewed study (Neck et al. 2026). The clinical symptoms monitored in our study may capture signs of neuroinvasion. Despite the low viral dose used to initiate infection, we acknowledge the possible occurrence of neuroinvasion, although we did not monitor viral load in the brain to confirm this point. Whether neutrophil aggregation also occurs in the brain requires further investigation, as the effect of anti‐Ly6G on clinical severity may in part reflect modulation of CNS‐related manifestations.

In contrast to neutrophils, lung‐associated classical monocytes exhibited a progressive macrophage differentiation associated with non‐severe disease, characterized by upregulation of PD‐L1, MHC‐II, CD64, and CXCR4. Unexpectedly, this differentiation began within the vascular compartment even before cMo infiltration into the parenchyma, as demonstrated by blood/tissue partitioning, but infiltrating cMo were proportionally more PD‐L1+, suggesting distinct functional roles within the lung tissue and the vasculature. This intravascular differentiation was strikingly reduced in adult mice developing severe clinical symptoms and even in aged mice regardless of clinical severity, suggesting an age‐related defect in cMo responses. Importantly, the frequency of PD‐L1^+^ classical monocytes inversely correlated with the neutrophil‐to‐monocyte ratio in the lungs, but not in circulation, underscoring the importance of local immune regulation at the site of infection (Sánchez‐Cerrillo et al. 2020; Cheon et al. 2021).

Unfortunately, we were not able to address the functional role of PD‐L1 specifically on monocytes. Our results support a compartment‐specific interpretation, whereby lung monocyte‐derived cells represent a distinct population that exhibits an activation profile characterized by I‐Ab, PD‐L1, CD64, and CXCR4 upregulation, which is associated with lower disease severity. In contrast, circulating classical monocytes do not exhibit the same association, underscoring the importance of tissue‐dependent and cell‐specific immune regulation rather than systemic PD‐L1 expression in shaping disease outcome. This observation may partially explain why anti‐PD‐L1 injections between day 4 and 8 post‐infection did not alter the course of the disease. In addition, it is noteworthy that anti‐PD‐L1 efficacy may also be dampened by soluble PD‐L1, which increases with COVID‐19 severity (Sabbatino et al. 2021; Trombetta et al. 2021). Specific neutralization of PD‐L1 expression on lung infiltrating cMo would be required to determine whether it represents a surrogate marker of monocyte activation or whether it exerts direct immunoregulatory functions to control the extent and duration of the response within the tissues.

Together, these findings reveal a compartmentalized myeloid program selectively engaged in protective pulmonary responses and decoupled from systemic immune signatures.

How these early events relate to long‐term tissue consequences remains unclear. Previous studies reported a higher accumulation of vimentin‐expressing CD68+ cells in the lung associated with persistent fibrosis and T‐cell dysregulation, particularly in elderly individuals (Lo Tartaro et al. 2022; Cheon et al. 2021). Our study focused on monocyte‐derived macrophages at early time points and not on lung resident macrophages. Whether these two subsets differentially contribute to long‐term deleterious effects remains to be addressed.

COVID‐19 severity in the elderly has been associated with numerous factors involved in the cross‐talk between innate and adaptive immune responses, such as NLR, GM‐CSF, CXCL10, CCL2, and IL‐1β (Poisson et al. 2024; Zinatizadeh et al. 2023). PD‐L1 expression on monocytes is classically viewed as immunosuppressive through inhibition of T‐cell activation; however, increasing evidence indicates that the functional consequences of PD‐L1 signaling are highly context‐dependent. While systemic upregulation of soluble PD‐L1 and *PD‐L1* transcript on circulating immune cells and infected epithelial cells in severe COVID‐19 has been associated with immune evasion, adaptive immune suppression, and disease severity (Sabbatino et al. 2021; Trombetta et al. 2021), PD‐L1 expression on myeloid cells can also reflect appropriate activation by inflammatory cues, particularly type I and type II interferons (Garcia‐Diaz et al. 2017; Bazhin et al. 2018). In viral infections, interferon‐driven PD‐L1 expression has been shown to limit excessive immunopathology while preserving antiviral defense, thereby correlating with tissue tolerance and resolution of inflammation (Barber et al. 2006; Shaabani et al. 2016). Altogether, these observations highlight the dual nature of PD‐1/PD‐L1 signaling in infectious disease.

Strikingly, aging was associated with a defect in PD‐L1 upregulation, despite increased monocyte accumulation, indicating a qualitative impairment rather than a quantitative deficiency in both mouse and human lungs. This defect was more linked to aging than to clinical severity. A similar defect in PD‐L1 expression was recently reported in human monocytes from elderly individuals restimulated in vitro with viral immune complexes, while the production of type I IFN was higher than in monocytes from younger individuals (Domitien Payet et al. 2025). In aged mice, the failure to induce PD‐L1 on lung monocytes could reflect defective interferon sensing or signaling (Guihot et al. 2020; Dorgham et al. 2021), a feature of SARS‐CoV infection (Spiegel et al. 2005; Zinatizadeh et al. 2023), coupled with pronounced neutrophil‐driven inflammation and lung vascular lesions (Barnes et al. 2020; Combadière et al. 2021; Hazeldine and Lord 2021). This interpretation is further supported by our human data showing neutrophil and monocyte accumulation in the lungs, with an age‐dependent decline in PD‐L1 expression on bronchoalveolar monocytes, which inversely correlated with both age and the lung NMR. According to the multivariate analysis of human blood, while NLR remains strongly associated with disease severity across age groups, NMR emerged as a marker of immune dysregulation predominantly in older patients. The significant interaction between NMR and age indicates that the relevance of this biomarker increases with aging. These findings support an age‐dependent prognostic role of inflammatory biomarkers in severe disease. However, these observations should be interpreted as reflecting the influence of aging on an imbalanced myeloid response, rather than demonstrating a direct functional role of PD‐L1.

These findings are particularly relevant in light of seminal studies demonstrating that a substantial fraction of patients with life‐threatening COVID‐19 harbor inborn errors in interferon signaling pathways (Zhang, Bastard, et al. 2020) or neutralizing autoantibodies against type I interferons (Bastard et al. 2020). Such anti‐interferon autoantibodies are markedly enriched in elderly individuals and have been causally linked to impaired antiviral immunity and severe disease (Bastard et al. 2021). Our data suggest that, beyond defective viral control, impaired interferon activity may also compromise the induction of PD‐L1+ monocytes in the lung. In this context, PD‐L1 expression should be interpreted as a marker of monocyte activation state rather than evidence of a defined protective regulatory function. Thus, loss of interferon signaling may simultaneously promote viral persistence, excessive neutrophil activation, and altered monocyte activation states, providing a unifying framework linking aging, interferon deficiency, and myeloid dysregulation in severe COVID‐19. Whether PD‐L1 expression by monocytes directly controls neutrophil accumulation and arrest in the lung microvasculature, or whether these processes are independently regulated, remains to be determined.

Collectively, our results from a SARS‐CoV‐2 infection model support the hypothesis that age‐related disease severity is associated with an altered myeloid response, characterized by excessive neutrophil accumulation leading to vascular obstruction, coupled with an impaired tissue‐dependent activation of monocytes, potentially mediated by defective interferon signaling. This imbalance favors immunopathology over immune regulation, providing a mechanistic framework for the disproportionate vulnerability of elderly individuals to severe disease. Accordingly, therapeutic strategies aimed at restoring interferon responsiveness, limiting pathological neutrophil arrest, or modulating monocyte activation states may represent promising approaches to mitigate severe acute viral infections, particularly in aging populations.

## Author Contributions

Investigation and formal analysis: S.M., C.G., E.W.‐D., F.L., A.B. Investigation: N.G., S.B., E.W.‐D., J.P., F.L., S.B., A.G., C.P., K.D. Provided resources: S.M., C.G., C.E., J.‐L.D., E.P., C.E.‐S., A.‐G.M., V.C., S.M., A.G., G.G., C.C., A.B. Writing – review and editing: S.M., C.G., A.B. Conceptualization and supervision: S.M., C.C., A.B. Project administration: S.M., C.C., A.B. Funding acquisition: S.M., C.C., A.B.

## Funding

This work was supported by Institut National de la Santé et de la Recherche Médicale (INSERM); Sorbonne Université; Agence Nationale de la Recherche (Grants ANR‐21‐CE44‐0030‐04 GET‐REDI, ANR‐20‐COV7‐0010‐01 and ANR‐21‐RHUS‐08) and Fondation pour la Recherche Médicale (Grant EQU202203014622).

## Conflicts of Interest

The authors declare no conflicts of interest.

## Supporting information


**Figure S1:** Kinetics of neutrophil and monocyte response during SARS‐CoV‐2 infection in the lungs. (A) Graphs represent the clinical severity score progression after SARS‐CoV‐2 inoculation in males (*n* = 18) and females (*n* = 21). (B) Viral titration by qPCR in lung tissue according to disease severity, 7 days after infection. Viral copies were undetected in 4 out of 32 mice tested. Data are pooled from three independent experiments. (C, D) Gating strategy for neutrophil, classical monocyte (cMo) and non‐classical monocyte (ncMo) identification in the blood (C) and the lungs (D). (E, F) Whisker plots show the number of indicated cells per mL of blood (E) and per total lung (F) at indicated time points after SARS‐CoV‐2 inoculation. Each dot represents one mouse. Data are pooled from at least two independent experiments per time point. Kruskal‐Wallis with Dunn's multiple comparison tests were performed. *p < 0.05; ***p < 0.001.
**Figure S2:** Monocytes and neutrophils aggregate in lung vasculature during SARS‐CoV‐2 infection. (A) Histogram plots show the fluorescent signature of neutrophils, classical (cMo) and non‐classical monocytes (ncMo) in the ACE2‐RGB‐Mac transgenic mouse strain according to Petit et al. (2024). Neutrophils are mApple+ GFP‐ ECFP‐ (R+), cMo are mApple+ GFPlow ECFP+ (RGB+), ncMo are mApple+ GFP+ ECFP+ (RGB+). (B) Wide field and (C) high magnification confocal images of lung cryosections from infected (day 6) or non‐infected ACE2‐RGB‐mac transgenic mouse show neutrophils (*R*+ cells) and monocytes (RGB+ cells) aggregate (white circle in B)) in the lung parenchyma and lung vasculature.
**Figure S3:** Circulating neutrophils and monocytes exhibit distinct activation profiles from the lungs. Representative flow cytometry dot plots and quantification of PD‐L1, I‐Ab, and CXCR4 expression on blood (A) neutrophils, (B) classical monocytes (cMo), (C) non‐classical monocytes (ncMo), according to the clinical status, 7 days after SARS‐CoV‐2 inoculation. Cells were identified based on the gating strategy in Figure S1. Kruskal‐Wallis with Dunn's multiple comparison tests were performed. Data are pooled from at least two independent experiments. *p < 0.05; **p < 0.01; ***p < 0.001; ****p < 0.0001. (D) Representative flow cytometry dot plots and quantification of PD‐L1 expression on lung infiltrating cells determined by blood/tissue partitioning, after intravascular injection of anti‐CD45‐PerCP‐Cy5.5 (CD45i.v.). Each dot indicates one mouse. Data are pooled from two independent experiments. Student *t* test was performed. **p < 0.01. (E) Representative dot plot showing PD‐L1 expression on circulating cMo (Ly6C+) and ncMo (Ly6C−) after anti‐PD‐L1 (black dots) or isotype control treatment (blue dots) 7 days after infection. Percent±SD of PD‐L1+ cMo and ncMo are indicated. (F) Kaplan–Meier survival curves after SARS‐CoV‐2 intranasal inoculation and treatment with 300 μg of anti‐PD‐L1 or isotype at day 4, 6, and 8. End‐point was considered when severity scoring reached 5. Data are pooled from three independent experiments, *n* = 25 females/group. (G) Quantification of the absolute number of neutrophils, cMo, and ncMo per mL of blood, 7 days after infection and treatment with anti‐PD‐L1 or isotype.
**Figure S4:** Age‐related COVID‐19 severity is associated with high NMR and a defect in monocyte activation in the lungs. (A) Whisker plots show the NMR in the blood (upper panels) and the lungs (lower panels) of old mice, according to the clinical status, 7 days after infection. Each dot represents one mouse. Kruskal‐Wallis with Dunn's multiple comparison tests were performed. *p < 0.05. (B, C) Representative dot plots show the co‐expression of CD64 and PD‐L1 on cMo according to their tissue localization: Vascular CD45i.v. + or infiltrating (CD45i.v‐) in adult (B) and old mice (C). Percent± SD of gated cells are indicated (*n* = 3 per group from one representative experiment).
**Figure S5:** NMR increases over time in critical COVID‐19 patients. Scatter plots show the (A) neutrophil (upper panel) and monocyte (lower panel) cell counts and (B) the neutrophil‐to‐lymphocyte ratio (NLR), according to clinical severity and age of the patients. Each dot represents one patient. Red dots indicate deceased patients in the following days. Data are extracted from the indicated publications. For patients < 70 years old, Mann–Whitney tests were performed. For patients > 70 years old, Kruskal‐Wallis with Dunn's multiple comparison tests were performed. **p < 0.01; ***p < 0.001; ****p < 0.0001. (C) Forest plot shows adjusted odds ratios (OR) and 95% confidence intervals (CI) from multivariate logistic regression analysis including continuous gender‐ and age‐biomarker interaction terms. All continuous variables were standardized as z‐scores. The dashed vertical line represents OR = 1, OR, 95% CI, and *p*‐value are indicated. Wald tests were performed. (D) Receiver operating characteristic (ROC) curves assess the discriminative performance of the indicated models. Age was modeled as a continuous variable. Patients from non‐severe and non‐critical groups were considered against patients from the critical group. DeLong tests were performed to assess AUC differences, *p* values are indicated. Analyzes in (C, D) were performed on the pooled cohort of patients < 70 (Kilercik et al. 2021) and ≥ 70 years (Poisson et al. 2024).
**Figure S6:** Neutrophils and monocytes aggregate in the lungs of critical COVID‐19 patients. Mass cytometry images of randomly selected lung regions from (A) three patients deceased from COVID‐19, previously published in Guihot et al. (2022) and (B) two patients deceased from cardiovascular diseases as controls, were analyzed for the relevant markers: CD14 (for classical monocyte identification), MPO (for neutrophil identification), CD68 (for macrophage identification), CD31 (for endothelial cell identification) and DNA (for whole tissue visualization). The totality of the imaged fields and patients generated from Guihot et al. (2022) are displayed.

**Video S1:** Live imaging of an explanted lung lobe shows neutrophil dynamics (*R*+ cells, red) in a non‐infected ACE2‐RGB‐Mac mouse. Representative track paths are indicated by colored dragon tails calculated using Imaris software. (RGB+ cells, cyan) represent monocytes and alveolar macrophages. (RG+ cells, green) represent interstitial macrophages. Lung tissue structures are detected by CARS imaging (gray).

**Video S2:** Live imaging of an explanted lung lobe shows neutrophil dynamics (*R*+ cells, red) in an ACE2‐RGB‐Mac mouse, 6 days after SARS‐CoV‐2 intranasal inoculation. Representative track paths are indicated by colored dragon tails calculated using Imaris software. (RGB+ cells, cyan) represent monocytes and alveolar macrophages. (RG+ cells, green) represent interstitial macrophages. Lung tissue structures are detected by CARS imaging (gray).

**Video S3:** Live imaging of an explanted lung lobe shows neutrophil dynamics (*R*+ cells, red) in an ACE2‐RGB‐Mac mouse, 6 days after SARS‐CoV‐2 intranasal inoculation in the presence of anti‐Ly6G. Representative track paths are indicated by colored dragon tails calculated using Imaris software. (RGB+ cells, cyan) represent monocytes and alveolar macrophages. (RG+ cells, green) represent interstitial macrophages. Lung tissue structures are detected by CARS imaging (gray).

## Data Availability

Some of the data that support the findings of this study are available on request from the corresponding author. Some data are derived from public domain resources.
