# Supplementary material for: Opposing Association of Lung Neutrophils and PD‐L1+ Monocytes in Age‐Related Severity of SARS‐CoV‐2 Infection
Source: Aging Cell. 2026 Jun 12;25(6):e70588. doi: 10.1111/acel.70588 (PMC13261690; doi:10.1111/acel.70588)
Supplement: Supplementary file 1 — Figure S1: Kinetics of neutrophil and monocyte response during SARS‐CoV‐2 infection in the lungs. (A) Graphs represent the clinical severity score progression after SARS‐CoV‐2 inoculation in males (n = 18) and females (n = 21). (B) Viral titration by qPCR in lung tissue according to disease severity, 7 days after infection. Viral copies were undetected in 4 out of 32 mice tested. Data are pooled from three independent experiments. (C, D) Gating strategy for neutrophil, classical monocyte (cMo) and non‐classical monocyte (ncMo) identification in the blood (C) and the lungs (D). (E, F) Whisker plots show the number of indicated cells per mL of blood (E) and per total lung (F) at indicated time points after SARS‐CoV‐2 inoculation. Each dot represents one mouse. Data are pooled from at least two independent experiments per time point. Kruskal‐Wallis with Dunn's multiple comparison tests were performed. *p < 0.05; ***p < 0.001. Figure S2: Monocytes and neutrophils aggregate in lung vasculature during SARS‐CoV‐2 infection. (A) Histogram plots show the fluorescent signature of neutrophils, classical (cMo) and non‐classical monocytes (ncMo) in the ACE2‐RGB‐Mac transgenic mouse strain according to Petit et al. (2024). Neutrophils are mApple+ GFP‐ ECFP‐ (R+), cMo are mApple+ GFPlow ECFP+ (RGB+), ncMo are mApple+ GFP+ ECFP+ (RGB+). (B) Wide field and (C) high magnification confocal images of lung cryosections from infected (day 6) or non‐infected ACE2‐RGB‐mac transgenic mouse show neutrophils (R+ cells) and monocytes (RGB+ cells) aggregate (white circle in B)) in the lung parenchyma and lung vasculature. Figure S3: Circulating neutrophils and monocytes exhibit distinct activation profiles from the lungs. Representative flow cytometry dot plots and quantification of PD‐L1, I‐Ab, and CXCR4 expression on blood (A) neutrophils, (B) classical monocytes (cMo), (C) non‐classical monocytes (ncMo), according to the clinical status, 7 days after SARS‐CoV‐2 inoculation. Cells were identi [file ACEL-25-e70588-s003.pdf]

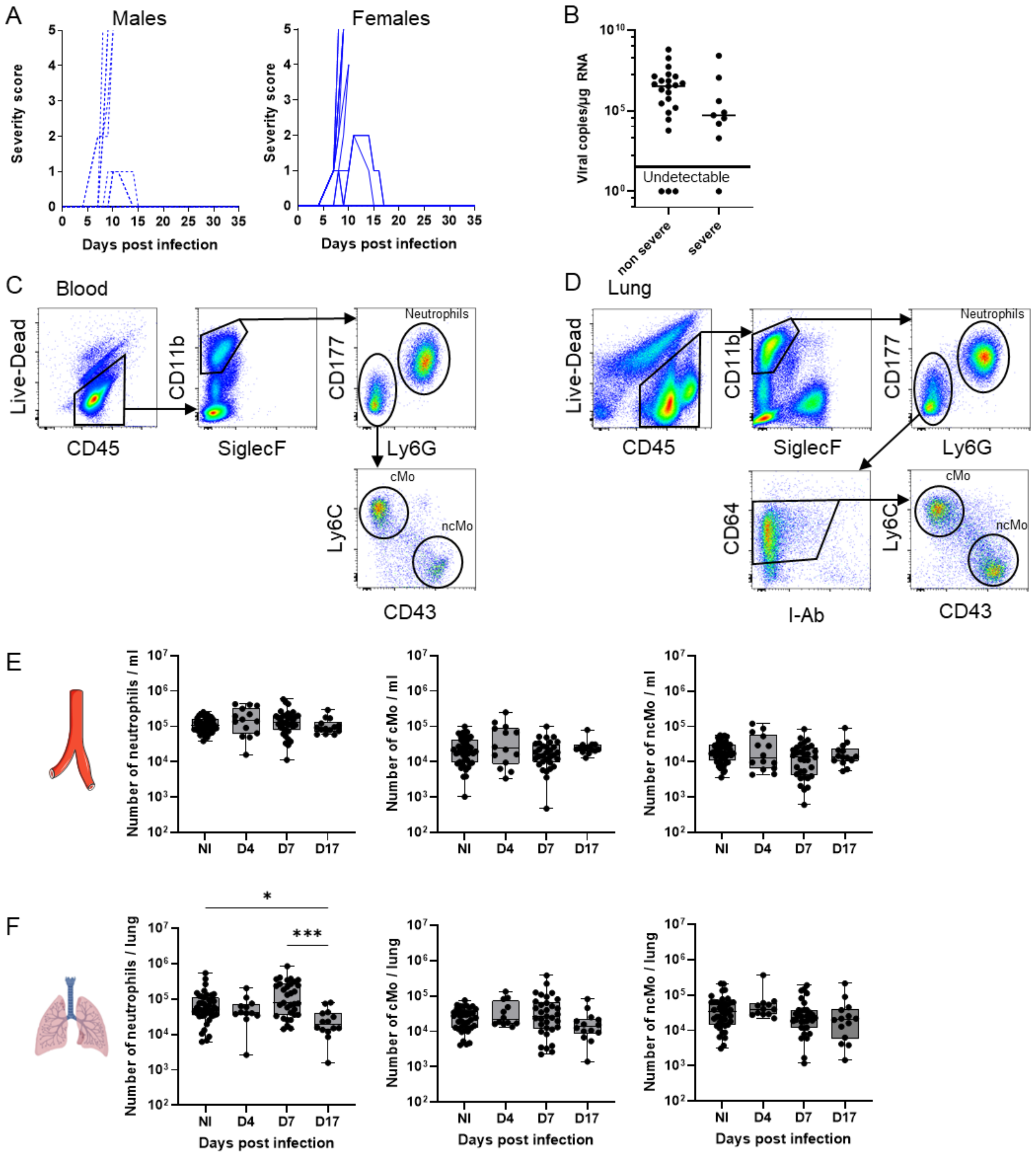

Figure S1. Kinetics of neutrophil and monocyte response during SARS-CoV-2 infection in the lungs

A) Graphs represent the clinical severity score progression after SARS-CoV-2 inoculation in males (n= 18) and females (n= 21). **B**) Viral titration by qPCR in lung tissue according to disease severity, 7 days after infection. Viral copies were undetected in 4 out of 32 mice tested. Data are pooled from three independent experiments. **C, D)** Gating strategy for neutrophil, classical monocyte (cMo) and non-classical monocyte (ncMo) identification in the blood **C**) and the lungs **D**). **E, F**) Whisker plots show the number of indicated cells per mL of blood **E**) and per total lung **F**) at indicated time points after SARS-CoV-2 inoculation. Each dot represents one mouse. Data are pooled from at least two independent experiments per time point. Kruskal-Wallis with Dunn's multiple comparison tests were performed. *, $p < 0.05$; ***, $p < 0.001$.

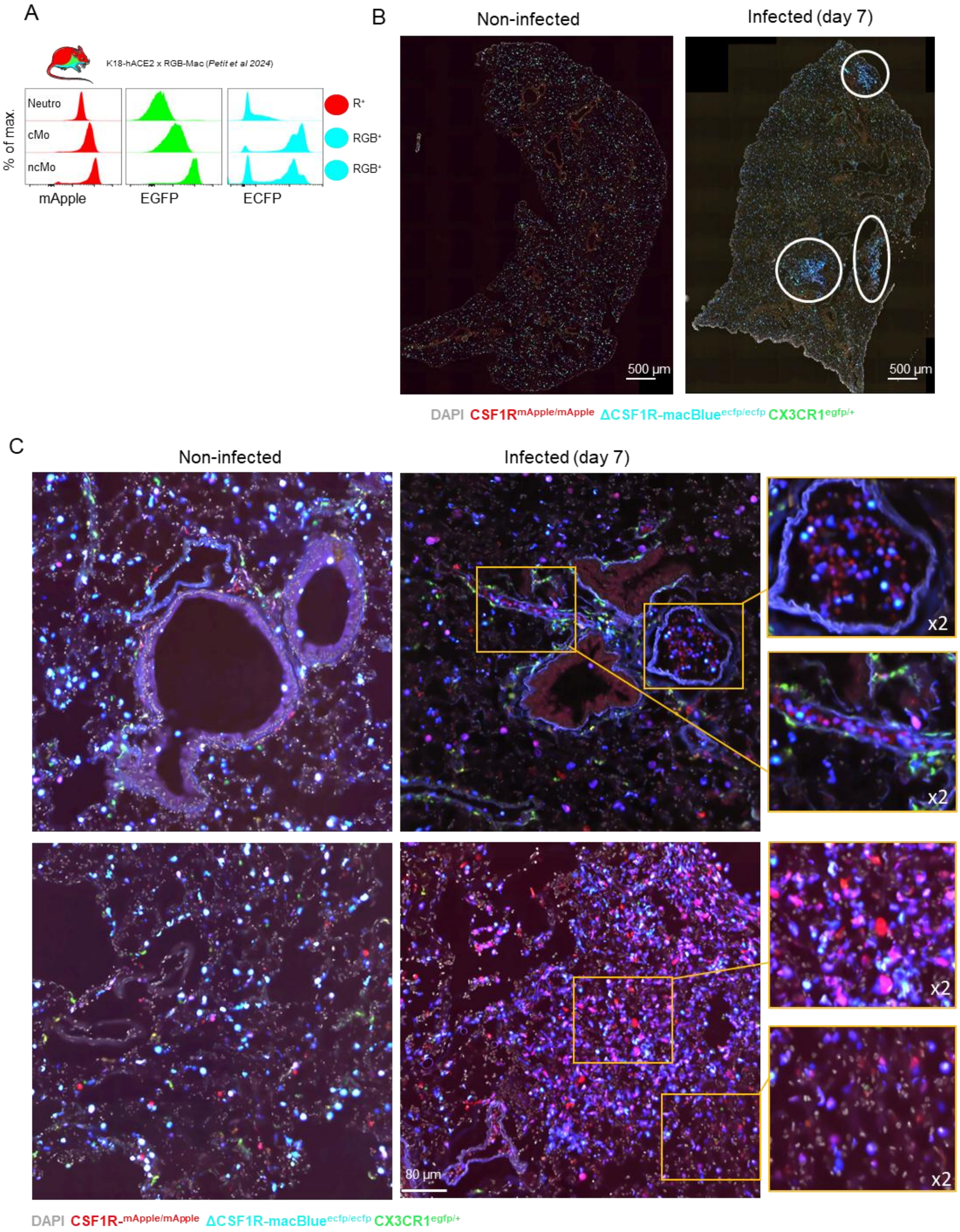

Figure S2. Monocytes and neutrophils aggregate in lung vasculature during SARS-CoV-2 infection

A) Histogram plots show the fluorescent signature of neutrophils, classical (cMo) and non-classical monocytes (ncMo) in the ACE2-RGB-Mac transgenic mouse strain according to (Petit et al. 2024). Neutrophils are mApple+ GFP- ECFP- (R+), cMo are mApple+ GFPlow ECFP+ (RGB+), ncMo are mApple+ GFP+ ECFP+ (RGB+). **B**) Wide field and **C**) high magnification confocal images of lung cryosections from infected (day 6) or non-infected ACE2-RGB-mac transgenic mouse show neutrophils (R+ cells) and monocytes (RGB+ cells) aggregate (white circle in **B**)) in the lung parenchyma and lung vasculature.

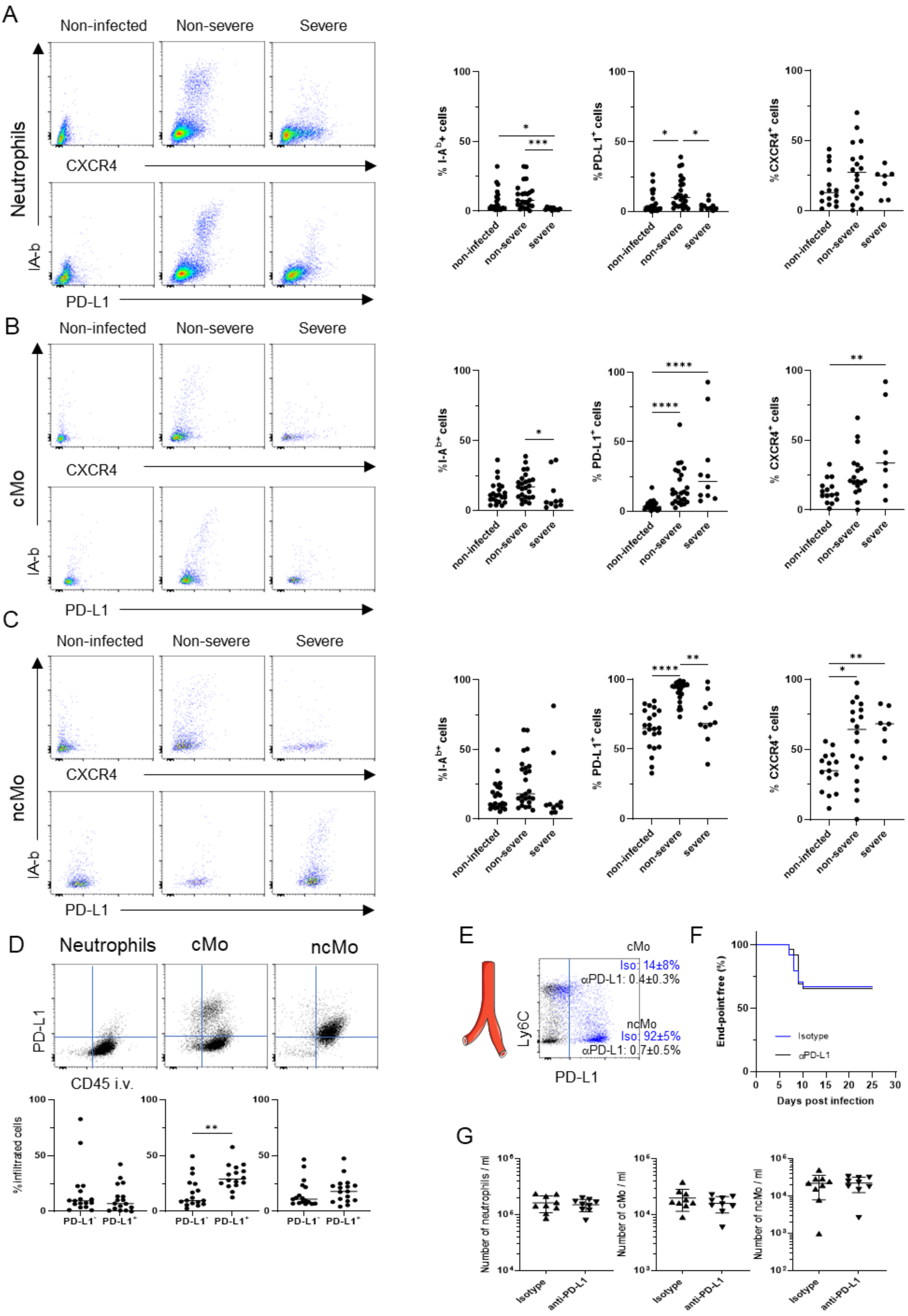
A
Non-infected
Non-severe
Severe
Neutrophils
CXCR4
IA-b
PD-L1
% I-A^b+ cells
% PD-L1$^+$ cells
% CXCR4$^+$ cells
non-infected
non-severe
severe
B
cMo
% I-A^{b+} cells
C
ncMo
D
Neutrophils
cMo
ncMo
PD-L1
CD45 i.v.
% infiltrated cells
PD-L1$^-$
PD-L1$^+$
E
cMo
Iso: 14±8%
αPD-L1: 0.4±0.3%
ncMo
Iso: 92±5%
αPD-L1: 0.7±0.5%
Ly6C
PD-L1
F
End-point free (%)
Isotype
αPD-L1
Days post infection
G
Number of neutrophils / ml
Number of cMo / ml
Number of ncMo / ml
Isotype
anti-PD-L1

Figure S3 Circulating neutrophils and monocytes exhibit distinct activation profiles from the lungs

Representative flow cytometry dot plots and quantification of PD-L1, I-Ab, and CXCR4 expression on blood **A**) neutrophils, **B**) classical monocytes (cMo), **C**) non-classical monocytes (ncMo), according to the clinical status, 7 days after SARS-CoV-2 inoculation. Cells were identified based on the gating strategy in F**igure S1**. Kruskal-Wallis with Dunn's multiple comparison tests were performed. Data are pooled from at least two independent experiments. *, $p < 0.05$; **, $p < 0.01$; ***, $p < 0.001$; ****, $p < 0.0001$. **D**) Representative flow cytometry dot plots and quantification of PD-L1 expression on lung infiltrating cells determined by blood / tissue partitioning, after intravascular injection of anti-CD45-PerCP-Cy5.5 (CD45i.v.). Each dot indicates one mouse. Data are pooled from two independent experiments. Student t-test was performed. **, $p < 0.01$. **E)** Representative dot plot showing PD-L1 expression on circulating cMo (Ly6C+) and ncMo (Ly6C-) after anti-PD-L1 (black dots) or isotype control treatment (blue dots) 7 days after infection. Percent±SD of PD-L1+ cMo and ncMo are indicated. **F**) Kaplan–Meier survival curves after SARS-CoV-2 intranasal inoculation and treatment with 300µg of anti-PD-L1 or isotype at day 4, 6, and 8. End-point was considered when severity scoring reached 5. Data are pooled from three independent experiments, n = 25 females / group. **G**) Quantification of the absolute number of neutrophils, cMo, and ncMo per mL of blood, 7 days after infection and treatment with anti-PD-L1 or isotype.

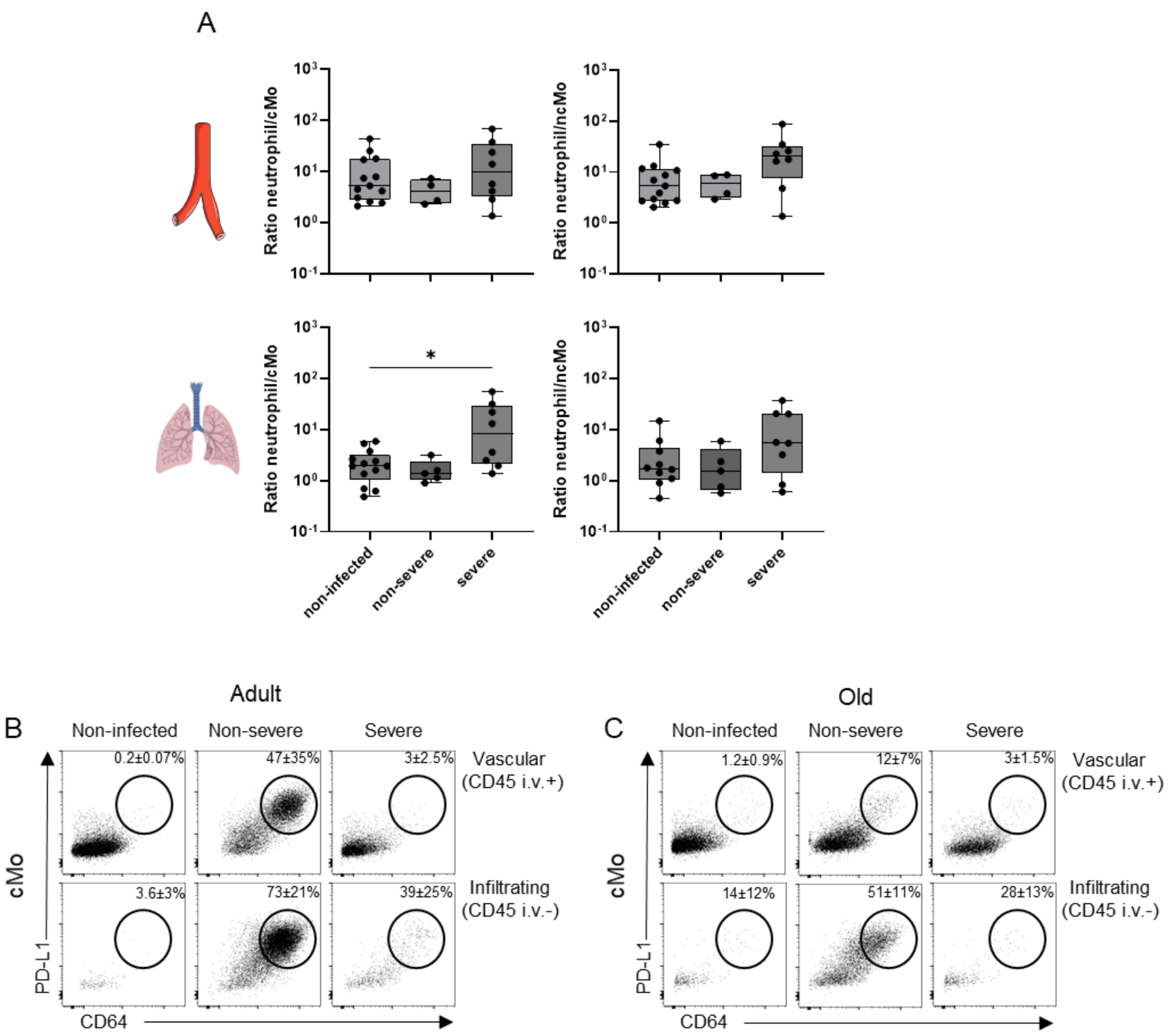

Sup Figure 4. Age-related COVID-19 severity is associated with high NMR and a defect in monocyte activation in the lungs

A) Whisker plots show the NMR in the blood (upper panels) and the lungs (lower panels) of old mice, according to the clinical status, 7 days after infection. Each dot represents one mouse. Kruskal-Wallis with Dunn's multiple comparison tests were performed. *, $p < 0.05$. **B-C**) Representative dot plots show the co-expression of CD64 and PD-L1 on cMo according to their tissue localization: Vascular CD45i.v.+ or infiltrating (CD45i.v-) in adult **B**) and old mice **C**). Percent± SD of gated cells are indicated (n=3 per group from one representative experiment).

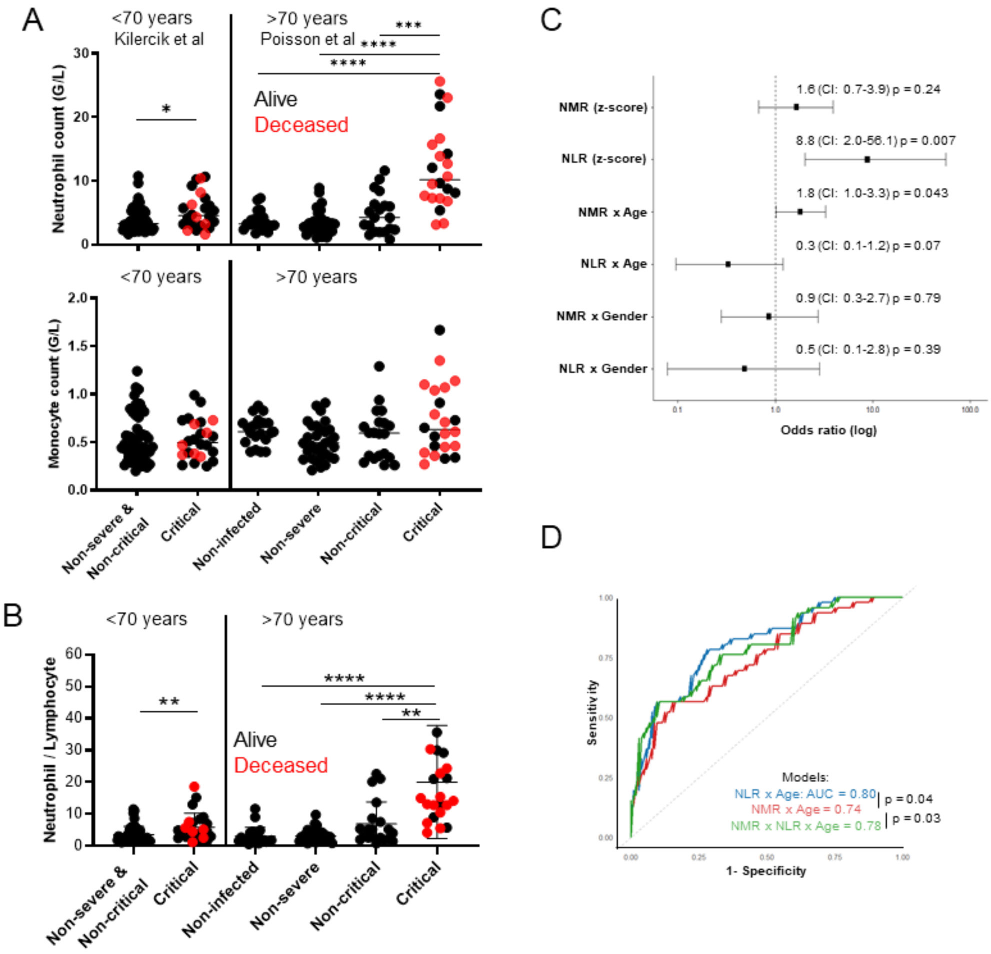

Figure S5 NMR increases over time in critical COVID-19 patients

Scatter plots show the **A**) neutrophil (upper panel) and monocyte (lower panel) cell counts and **B**) the neutrophil-to-lymphocyte ratio (NLR), according to clinical severity and age of the patients. Each dot represents one patient. Red dots indicate deceased patients in the following days. Data are extracted from the indicated publications. For patients < 70 years old, Mann-Whitney tests were performed. For patients > 70 years old, Kruskal-Wallis with Dunn's multiple comparison tests were performed. **, p < 0.01; ***, p < 0.001; ****, p < 0.0001. **C**) Forest plot shows adjusted odds ratios (OR) and 95% confidence intervals (CI) from multivariate logistic regression analysis including continuous gender- and age-biomarker interaction terms. All continuous variables were standardized as z-scores. The dashed vertical line represents OR = 1, OR, 95% CI, and p-value are indicated. Wald tests were performed. **D**) Receiver operating characteristic (ROC) curves assess the discriminative performance of the indicated models. Age was modeled as a continuous variable. Patients from non-severe and non-critical groups were considered against patients from the critical group. DeLong tests were performed to assess AUC differences, p values are indicated. Analyses in **C-D**) were performed on the pooled cohort of patients <70 (Kilercik et al) and ≥70 years (Poisson et al).

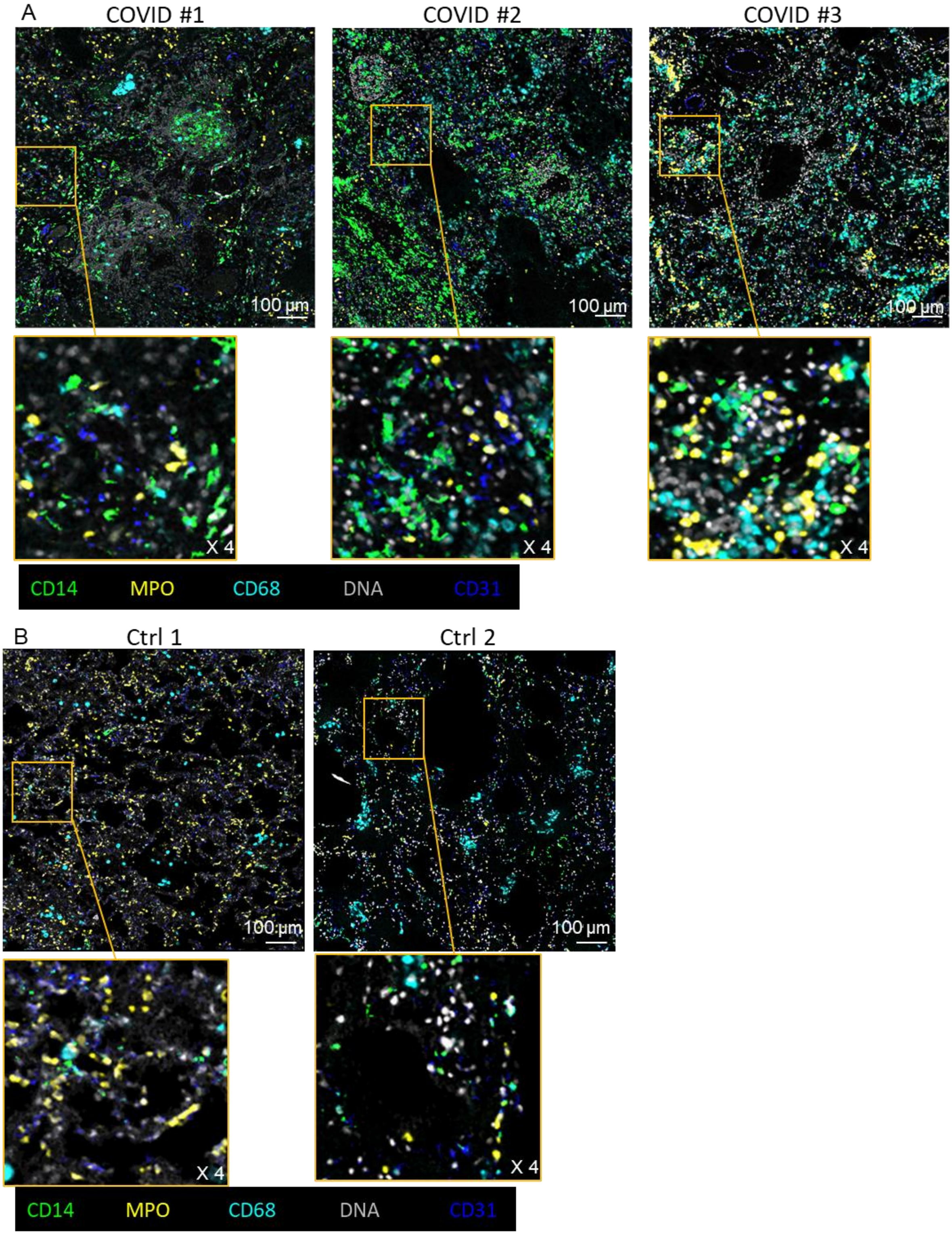

Figure S6 Neutrophils and monocytes aggregate in the lungs of critical COVID-19 patients

Mass cytometry images of randomly selected lung regions from **A**) three patients deceased from COVID-19, previously published in (Guihot et al. 2022) and **B**) two patients deceased from cardiovascular diseases as controls, were analyzed for the relevant markers: CD14 (for classical monocyte identification), MPO (for neutrophil identification), CD68 (for macrophage identification), CD31 (for endothelial cell identification) and DNA (for whole tissue visualization). The totality of the imaged fields and patients generated from (Guihot et al. 2022) are displayed.

Supplementary video and legends

Videos can be downloaded from: https://drive.google.com/drive/folders/11wN-HV6RYDZC4rHlhty14Mbif0f8YqaV?usp=sharing

Video1 Live imaging of an explanted lung lobe shows neutrophil dynamics (R+ cells, red) in a non-infected ACE2-RGB-Mac mouse. Representative track paths are indicated by colored dragon tails calculated using Imaris software. (RGB+ cells, cyan) represent monocytes and alveolar macrophages. (RG+ cells, green) represent interstitial macrophages. Lung tissue structures are detected by CARS imaging (gray)

Video 2 Live imaging of an explanted lung lobe shows neutrophil dynamics (R+ cells, red) in an ACE2-RGB-Mac mouse, 6 days after SARS-CoV-2 intranasal inoculation. Representative track paths are indicated by colored dragon tails calculated using Imaris software. (RGB+ cells, cyan) represent monocytes and alveolar macrophages. (RG+ cells, green) represent interstitial macrophages. Lung tissue structures are detected by CARS imaging (grey)

Video 3 Live imaging of an explanted lung lobe shows neutrophil dynamics (R+ cells, red) in an ACE2-RGB-Mac mouse, 6 days after SARS-CoV-2 intranasal inoculation in the presence of anti-Ly6G. Representative track paths are indicated by colored dragon tails calculated using Imaris software. (RGB+ cells, cyan) represent monocytes and alveolar macrophages. (RG+ cells, green) represent interstitial macrophages. Lung tissue structures are detected by CARS imaging (grey).
